# Automatic Detection of Adverse Drug Events in Geriatric Care: Study Proposal

**DOI:** 10.2196/40456

**Published:** 2022-11-15

**Authors:** Frederic Gaspar, Monika Lutters, Patrick Emanuel Beeler, Pierre Olivier Lang, Bernard Burnand, Fabio Rinaldi, Christian Lovis, Chantal Csajka, Marie-Annick Le Pogam, SwissMADE study

**Affiliations:** 1 Center for Research and Innovation in Clinical Pharmaceutical Sciences Lausanne University Hospital and University of Lausanne Lausanne Switzerland; 2 School of Pharmaceutical Sciences, University of Geneva Geneva Switzerland; 3 Institute of Pharmaceutical Sciences of Western Switzerland, University of Geneva, University of Lausanne Geneva and Lausanne Switzerland; 4 Service of Clinical Pharmacy Baden University Hospital Baden Switzerland; 5 Division of Occupational and Environmental Medicine, Epidemiology, Biostatistics and Prevention Institute University of Zurich and University Hospital Zurich Zurich Switzerland; 6 Clinique de Montchoisi Lausanne Switzerland; 7 Unisanté Center for Primary Care and Public Health Department of Epidemiology and Health Systems University of Lausanne Lausanne Switzerland; 8 Dalle Molle Institute for Artificial Intelligence Research Scuola Universitaria Professionale della Svizzera Italiana Universita della Svizzera Italiana Lugano Switzerland; 9 Department of Quantitative Biomedicine University of Zurich Zurich Switzerland; 10 Swiss Institute of Bioinformatics Lausanne Switzerland; 11 Fondazione Bruno Kessler Trento Italy; 12 Division of Medical Information Sciences Geneva University Hospitals and University of Geneva Geneva Switzerland; 13 See Acknowledgments

**Keywords:** adverse drug events, adverse drug reactions, older inpatients, aged 65 and older, multimorbidity, polypharmacy, patient safety, inappropriate prescribing, medication errors, natural language processing, clinical decision support system, automated adverse drug event reporting system, electronic medical record, hospitals, multicenter study, interdisciplinary research, quality of hospital care, machine learning, antithrombotics, venous thromboembolism, hemorrhage

## Abstract

**Background:**

One-third of older inpatients experience adverse drug events (ADEs), which increase their mortality, morbidity, and health care use and costs. In particular, antithrombotic drugs are among the most at-risk medications for this population. Reporting systems have been implemented at the national, regional, and provider levels to monitor ADEs and design prevention strategies. Owing to their well-known limitations, automated detection technologies based on electronic medical records (EMRs) are being developed to routinely detect or predict ADEs.

**Objective:**

This study aims to develop and validate an automated detection tool for monitoring antithrombotic-related ADEs using EMRs from 4 large Swiss hospitals. We aim to assess cumulative incidences of hemorrhages and thromboses in older inpatients associated with the prescription of antithrombotic drugs, identify triggering factors, and propose improvements for clinical practice.

**Methods:**

This project is a multicenter, cross-sectional study based on 2015 to 2016 EMR data from 4 large hospitals in Switzerland: Lausanne, Geneva, and Zürich university hospitals, and Baden Cantonal Hospital. We have included inpatients aged ≥65 years who stayed at 1 of the 4 hospitals during 2015 or 2016, received at least one antithrombotic drug during their stay, and signed or were not opposed to a general consent for participation in research. First, clinical experts selected a list of relevant antithrombotic drugs along with their side effects, risks, and confounding factors. Second, administrative, clinical, prescription, and laboratory data available in the form of free text and structured data were extracted from study participants’ EMRs. Third, several automated rule-based and machine learning–based algorithms are being developed, allowing for the identification of hemorrhage and thromboembolic events and their triggering factors from the extracted information. Finally, we plan to validate the developed detection tools (one per ADE type) through manual medical record review. Performance metrics for assessing internal validity will comprise the area under the receiver operating characteristic curve, *F*_1_-score, sensitivity, specificity, and positive and negative predictive values.

**Results:**

After accounting for the inclusion and exclusion criteria, we will include 34,522 residents aged ≥65 years. The data will be analyzed in 2022, and the research project will run until the end of 2022 to mid-2023.

**Conclusions:**

This project will allow for the introduction of measures to improve safety in prescribing antithrombotic drugs, which today remain among the drugs most involved in ADEs. The findings will be implemented in clinical practice using indicators of adverse events for risk management and training for health care professionals; the tools and methodologies developed will be disseminated for new research in this field. The increased performance of natural language processing as an important complement to structured data will bring existing tools to another level of efficiency in the detection of ADEs. Currently, such systems are unavailable in Switzerland.

**International Registered Report Identifier (IRRID):**

DERR1-10.2196/40456

## Introduction

### Adverse Drug Events in Older Inpatients: A Significant Health Issue

Patient injury resulting from medication use [1,2], also known as adverse drug events (ADEs), is the second most frequent complication experienced by hospitalized patients, accounting for approximately one-third (10%-40%) of all inpatient care-related adverse events [3,4]. ADEs include nonpreventable ADEs subsequent to appropriate care and preventable ADEs (pADEs) resulting from suboptimal care [5]. Between 0.2% and 65% of hospitalized patients experience at least one ADE during their stay [5-9]. This prevalence depends on the selected definition of ADEs, the methods used for their detection, the study size, various risk factors related to the clinical setting (eg, medical or surgical), patient characteristics (eg, age of ≥65 years and polypharmacy), and prescribed drugs [6,8,10,11]. Apart from increasing morbidity and mortality, ADEs have a significant impact on hospital use (ie, increased length of stay and readmissions) and associated costs [2,4,12-15]. Moreover, pADEs, which account for 20% to 50% of all ADEs, are often more serious and associated with increased lengths of stay and costs compared with non-pADEs [2,4,8,13,15].

Older inpatients (aged ≥65 years) and, in particular, oldest-old inpatients (aged ≥80 years) are especially at risk of ADEs and pADEs. Over 30% of these patients experience at least one ADE during their hospital stay, and up to 70% of these events are deemed preventable [8,16-18]. In addition, ADEs have more severe consequences in this population, such as inducing or worsening frailty; causing functional and cognitive disability; and leading to loss of autonomy, more frequent and prolonged hospitalizations, nursing home admissions, and even death [16,17,19]. Finally, ADEs affect patients’ hospital care experience and quality of life [19,20]. Given that older patients are hospitals’ most frequent users [21,22], ADEs represent an important clinical and economic burden to this population and to health systems [23,24]. Thus, limiting ADEs has become a major patient safety and public health concern worldwide [23].

### Antithrombotic Therapy

Cardiovascular drugs, in particular antithrombotic and antihypertensive drugs, are frequently associated with ADEs in older patients. Although recommended and widely used in older patients who are at increased risk of cardiovascular events, antiplatelet and anticoagulant treatments are highly associated with bleeding complications in this population and are a major cause of emergency department admissions and death [25-27]. Thus, antithrombotic therapy is similar to the sword of Damocles, conferring protection against thrombosis while exposing patients to bleeding, with severe consequences in both cases [27]. A recent study indicated that bleeding events were the most common ADE (36%) in patients aged >65 years [28]. The drugs most frequently involved in serious ADEs were antithrombotic agents (31%). Disregarding drug interactions, contraindications, and precautions caused 20% of ADEs, and drug overdoses were present in 17% [28]. In addition, combinations of factors and inefficacy raise particular concerns from an individual and public health point of view [28].

### Epidemiology and Risk Management of ADEs in Swiss Inpatients

The Swiss health system is taking an increasing interest in medication safety issues. Few studies have been conducted in Switzerland to assess the incidence of ADEs during hospital stays and ADEs as the cause of hospital admissions [29-32]. A cohort study (Stiftung für Arzneimittelsicherheit or Comprehensive Hospital Drug Monitoring) conducted in the internal medicine departments of Zürich and St. Gallen university hospitals found incidences of ADEs and pADEs of 11.2% and 0.4%, respectively [30,31]. The causative drugs were antithrombotic and cardiovascular drugs, antibiotics, hypnotics, and nonsteroidal anti-inflammatory drugs. The observed pADE-related mortality was 3 deaths per 100,000 persons annually [30,31]. Another study conducted in 10 hospitals in the canton of Vaud estimated that between 10% and 17% of hospitalized patients were exposed annually to ADEs [32]. In this study, a patient safety improvement program was deployed over 18 months with the aim of reducing ADEs by 20%. After implementing the patient safety program, which entailed patient identification, high-alert medication, and medication preparation in the ward, the annual rate of harmed patients decreased to 7% within 2 years [32]. Another study conducted at Lausanne University Hospital (LUH) found that 7% of all emergency department admissions were caused by ADEs, of which 32% were classified as avoidable. The most frequent ADEs were gastrointestinal bleeding (22.3%) and febrile neutropenia (14.4%) [29]. Similar results were reported in a prospective analysis of reasons for hospital admission in the internal medicine department of the Bellinzona Regional Hospital. The authors estimated that 6.4% of patients admitted over 1 year presented with ADEs at admission and that most of them were potentially preventable. Cardiovascular and cerebrovascular drugs accounted for 65% of ADEs, and the risks of occurrence increased with age, polypharmacy, multimorbidity, and length of stay. The authors finally estimated that, in Switzerland, 12,000 to 16,000 admissions per year were caused by inappropriate or unnecessary treatment, with additional direct annual costs of CHF 70 to CHF 100 million (US $70.8-101.1 million) [33].

### ADE Detection Tools

Worldwide, numerous optimization strategies have been adopted to improve the quality and safety of medications prescribed to older patients [4,23,34-37]. Among these are evidence-based specific guidelines [38]; lists of potentially inappropriate medication criteria [37,39]; pharmacist-based interventions, including patient counseling [35]; medication reconciliation; clinical pharmacist rounding; and team-based interventions such as multidisciplinary geriatric teams [35,36,40,41].

The availability of clinical decision support systems within computer provider order entry systems has raised hopes and expectations to improve the safety and efficiency of care [42]. Clinical decision support systems comprise a wide range of functionalities, including medication dosing support, point-of-care alerts or reminders (eg, for drug-drug interactions), or workflow support for medication reconciliation [43-45]. Many studies have reported the positive impacts of such systems on patient outcomes, such as fewer duplicate orders, dosage errors, drug interactions, or delayed actions using reminders [41].

Clinical event monitors are a type of clinical information system with considerable potential to contribute to the detection and monitoring of medication-related problems, in particular ADEs [44]. Such systems provide feedback to clinicians through alerts and reminders when certain signals regarding pharmacy orders (eg, sudden stop orders, antidote ordering, and dose correction orders), laboratory test results, or patient characteristics are triggered [42]. Classen et al [43] developed a computerized method for detecting ADEs that uses signals identified from several types of patient medical record data. This computerized monitor increased >60-fold the detection and reporting of ADEs in hospitalized patients. Owing to growing evidence regarding the benefits of such clinical event monitors, several prominent national organizations have recommended their use to detect ADEs. Compared with voluntary reporting or manual methods of chart reviews, electronic clinical event monitors are faster, less expensive, and often identify ADEs that are not normally detected during the course of routine hospital care [6,44]. However, current clinical event monitors generate many false-positive alerts, target rather inappropriate prescriptions instead of clinically relevant ADEs, and do not consider the type of hospital or unit (eg, medical or surgical) or the patients’ characteristics [42,46,47]. In Switzerland, health care facilities have progressively introduced clinical decision support. However, current systems are mostly decision support tools targeting drug dosage or drug-drug interaction or incompatibilities or supporting information from the hospital drug formulary [40,46].

Owing to the poor specificity and overalerting of existing ADE detection tools and the availability of large amounts of structured and unstructured information contained in computer-based patient records, new ADE detection and monitoring systems are currently being developed [47-51]. They are based on multiple sources of data and rely on new methodologies for data processing combining structured data mining (SDM) and natural language processing (NLP). SDM is defined as the process of finding and extracting useful information from semistructured data, whereas NLP is a domain at the crossroads of computer science and linguistics that aims at modeling language to extract meaningful information from free text. Typically, structured data include drug names, doses, treatment durations, administration routes, laboratory results, and diagnostic or procedure codes, whereas reasons for admission, patient histories and conditions, nursing and medical progress notes, inpatient reports, and discharge summaries are essentially available in the form of free text. The use of NLP improves the ability to detect ADEs because the available data sources are often in free text. As a result, structured data analytics have the advantage of being language-independent, though limited by poor specificity and overalerting, whereas free-text analytics have strong power to support ADE detection while strongly depending on language [51]. Tools have already been developed for the English language but are not directly applicable to other languages such as French, German, or Italian, which are 3 of the 4 official languages in Switzerland. As a result, no ADE detection and monitoring system based on electronic medical record (EMR) SDM and NLP is currently available in Switzerland.

### Aim and Research Questions

This Swiss national initiative aims to develop and validate a multimodal, multisource, and multicentric approach for the automated detection of antithrombotic-related ADEs and their risk management in older inpatients. Our hypothesis is that the automated detection of ADEs from EMRs using SDM and NLP could significantly improve risk management and patient safety in hospitalized older inpatients with multimorbidity, frailty, and polypharmacy. This will provide reliable data regarding the incidence of ADEs for health care professionals, patient safety organizations, and policy makers. Thus, the project will comprise complementary steps aimed at (1) quantifying the cumulative incidence of ADEs associated with and caused by antithrombotic drugs; (2) assessing the causality, severity, and preventability of detected ADEs induced by antithrombotic drugs; and (3) developing strategies for the implementation of the project results to improve the risk management of antithrombotic drugs in the hospital setting.

## Methods

### Overall Design

We will conduct a multicenter cross-sectional study using retrospective medical data (years 2015 and 2016) from the EMRs of 4 large Swiss hospitals. Our project will include 2 hospitals in the French-speaking part of Switzerland (Lausanne and Geneva; LUH and Geneva University Hospital [GUH]) and 2 hospitals in the German-speaking part (Zürich and Baden; Zürich University Hospital [USZ] and Baden Cantonal Hospital [KSB]). Three are large university hospitals (GUH, LUH, and USZ), and one is a smaller cantonal hospital (KSB).

### Study Participants

Study participants will consist of all Swiss residents aged ≥65 years who were admitted for >24 hours to 1 of the 4 hospitals between 2015 and 2016 (ie, the inclusion period) and received at least one antithrombotic drug during their stay. We will exclude any patients for whom an explicit refusal to be involved in research projects or give access to their personal health data is documented.

### Source Data

This project will use health-related information that is routinely collected during daily practice. Relevant health-related data, as defined in this section, will be extracted by each participating hospital for all patient stays fulfilling the inclusion criteria.

Health-related data include the following: (1) structured data, which encompass each included stay; patient administrative data (eg, age, gender, place of residence, and admission and discharge mode and date); admission unit, diagnosis, and procedure codes (International Classification of Diseases, 10th Revision [ICD-10], and Swiss classification of surgical interventions [CHOP] codes); drug administration orders (eg, drug names, administration dates and times, drug dosages, frequencies, durations, administration routes, and Anatomical Therapeutic Chemical codes); laboratory test orders (ie, test names, time stamps, and samples); laboratory test results (ie, test names, typical ranges, units, results, and time stamps); and imaging and endoscopic test orders (ie, test names and time stamps); and (2) free-text data, including discharge letters, medical and nursing progress notes, pharmacological consultation notes, ADE reports, and all existing local metadata. The extracted variables are presented in Table 1 for structured data. The documents that will be extracted as free text are presented in Textbox 1. Details of the extracted items are presented in Tables S1-S4 in Multimedia Appendix 1.

**Table 1:** Structured data extracted for the project.

Data type, extracted data, and subcategory				Unit
**General administrative data**				
	Patient identification number (coded)			Category
	Case identification number (admission ID, hospitalization ID, or stay ID)			Category
	Insurance type			Category
	Region of residence (MedStat^a^ region)			Category
	Admission mode (eg, admission via emergency department, planned admission, or transfer)			Category
	Nationality			Category
	Date of birth (coded)			Date
	Gender			Category
	Date of death (if applicable)			Date
**Clinical measurements**				
	Blood pressure			Value
	Weight			Value
	Height			Value
	Sum of alcohol withdrawal syndrome score			Value
**Patient location or locations and transfers**				
	Unit of hospitalization			Category
	Transfers (medicine, surgery, intermediate care, and intensive care)			Category
	Date and time of admission			Date and time
	Date and time of discharge			Date and time
**Diagnoses and procedures**				
	DRG^b^ codes			Category
	CHOP^c^ codes			Category
	ICD-10^d^ codes			Category
	Readmissions and reasons for readmissions (first, second, third, fourth, and subsequent readmissions)			Category
	Drugs coded for reimbursement			Category
	Intensive care unit length of stay (in hours)			Category
	Duration of mechanical ventilation (in hours)			Category
	Disease severity and scores			Category
	NEMS^e^			Category
**Laboratory values^f^**				
	**Electrolytes and ions**			
		Blood ionogram (sodium and potassium)	mmol/L	
		Serum lactate and bicarbonate levels	mmol/L	
		Serum uric acid level	mmol/L	
		Serum urea level	mmol/L	
		Serum iron, transferrin saturation, and serum ferritin level	mmol/L and µg/L	
	**Enzymes**			
		Serum levels of aminotransferases	AST^g^ and ALT^h^ (UI^i^/L)	
		Serum levels of 5′-nucleotidase	UI/L	
		Serum level of CK^j^	UI/L	
		Serum level of GGT^k^	UI/L	
		Serum level of ALP^l^	UI/L	
	**CBC^m^**			
		Red blood cell count	Absolute value/mm^3^	
		Hemoglobin	mmol/L	
		Hematocrit	Percentage of total blood volume	
		Mean corpuscular volume	*µ* ^3^	
		White blood cell count	Absolute value/mm^3^	
		Platelet count	Absolute value/mm^3^	
		Reticulocyte count	Absolute value/mm^3^	
	**Hemostasis**			
		PT^n^	Time	
		APTT^o^	Time	
		TT^p^	Time	
		INR^q^	N/A^r^	
		Plasma fibrinogen	g/L	
		Procoagulant balance	Antithrombin (g/L), protein C and S (nmol/L), anticardiolipin antibody (GPL^s^ unit), and anti-beta-2-glycoprotein 1 antibody (GPL unit)	
		Individual coagulation factors	Percentage relative to a reference pool	
		Fibrinolysis	D-dimer (µg/L)	
		Anticoagulation monitoring	Anti-Xa (percentage relative to a reference value) and anti-IIa (percentage relative to a reference value)	
		Markers of coagulation	TAT^t^ (ng/mL) and fragment 1 and 2 of prothrombin (percentage relative to a reference value)	
	**Other**			
		Serum albumin	g/dL	
		Serum total protein	g/dL	
		Oxygen saturation in arterial blood	Percentage	
		CRP^u^	mg/L	
		Serum myoglobin	µg/L	
		Serum troponin	µg/L	
		Serum creatinine and creatinine clearance	mg/L and mL/minute	
		Serum total bilirubin and direct and indirect bilirubin	mg/L	
		Serum glycated hemoglobin	Percentage	
		Serum tumor markers available	N/A	
**Prescription or medication^v^**				
	ATC^w^ code (and product ID)			Category
	Information on dose and planned administration frequency, including unit (eg, milligrams)			Value and category
	Information on administration route (eg, intravenous administration vs oral)			Category
	PRN^x^ orders (“as needed”: drugs available at patient’s request; eg, analgesics)			Category
	Administrations performed (signed by nurses)			Category

^a^MedStat: MedStat regions are geographic areas with a sufficiently large population to anonymously assign a residence to each person hospitalized in Switzerland.

^b^DRG: Swiss Diagnosis Related Groups.

^c^CHOP: Swiss classification of surgical interventions.

^d^ICD-10: International Classification of Diseases, 10th Revision.

^e^NEMS: Nine Equivalents of Nursing Manpower Use Score.

^f^Laboratory results that were ordered or received within the time frame of any of the recorded or extracted stays (if available).

^g^AST: Aspartate aminotransferase.

^h^ALT: Alanine transaminase.

^i^UI: international unit.

^j^CK: creatine kinase.

^k^GGT: gamma-glutamyltransferase.

^l^ALP: alkaline phosphatase.

^m^CBC: complete blood count.

^n^PT: prothrombin time.

^o^APTT: activated partial thromboplastin time.

^p^TT: thrombin time.

^q^INR: international normalized ratio.

^r^N/A: not applicable.

^s^GPL: immunoglobulin G [IgG] phospholipid unit.

^t^TAT: thrombin-antithrombin III complex.

^u^CRP: C-reactive protein.

^v^All medication orders that have (1) a planned start date ≤discharge date AND (2) a planned discontinuation date ≥admission date.

^w^ATC: Anatomical Therapeutic Chemical.

^x^PRN: Pro re nata.

**Free texts and narratives extracted for the project.**

**Extracted free-text data**

- Patient identification number (metadata)
- Case identification number (admission ID, hospitalization ID, or stay ID, metadata)
- Notes taken at admission
- Discharge summaries and letters
- Nurses’ progress notes
- Imaging and radiology reports
- Specialists’ (eg, hematologist, cardiologist, angiologist, and particularly endoscopy reports) consultation notes
- Clinical pharmacology or pharmacy service consultation notes
- Adverse drug event or pharmacovigilance reports
- Critical incidence reporting system reports

### Data Analysis

**Figure 1:**
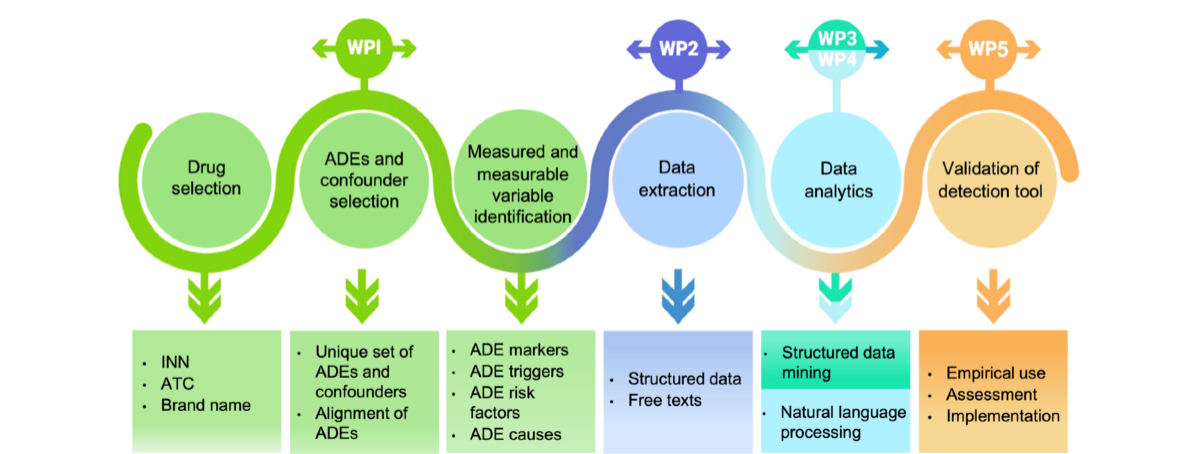
Schematic framework of the SwissMADE project. ATC: Anatomical Therapeutic Chemical; INN: international nonproprietary name; work package (WP) 1: drug selection and adverse drug event (ADE) marker and trigger definition and selection; WP 2: data extraction and management; WP 3: algorithm generation based on structured data; WP 4: resource and algorithm generation for free-text data; WP 5: ADE detection tool assessment.

The overall strategy includes the development of automated detection tools that will provide quantitative measures and triggers of ADEs associated with antithrombotic drugs and the elaboration of measures for implementation, replication, and prevention (Figure 1). A total of 5 work packages (WPs) will provide answers to these objectives.

### Drug Selection and ADE Marker or Trigger Definition and Selection From the Literature (WP 1)

Five different classes of antithrombotic drugs (approved and licensed in Switzerland) will be considered: heparins (eg, unfractionated heparins and low–molecular-weight heparins), vitamin K antagonists (eg, coumarin derivatives), directly acting oral anticoagulants and fondaparinux (eg, direct thrombin and factor Xa inhibitors), and antiplatelet drugs. The list of selected drugs is provided in Table S4 in Multimedia Appendix 1. For each administration of an antithrombotic drug during each inpatient stay, we will retrieve the following information from the corresponding EMR: international drug name; active pharmaceutical ingredient; Anatomical Therapeutic Chemical code; approved therapeutic regimen (eg, dose, range, frequency, and duration); pharmacokinetics (eg, absorption, primary metabolism pathways, and elimination); and potential drug-drug or drug-disease interactions, indications, and contraindications.

To identify thromboembolic and hemorrhagic ADEs caused by antithrombotics, we will first develop direct and indirect indicators of ADEs (Textbox 2). Potential clinical and biological antithrombotic-related ADEs will be assembled from clinical guidelines and standard pharmacological references, including Swiss medic product information, Lexicomp, Martindale, and Meyler’s Side Effects of Drugs encyclopedia [52]. We will focus on 3 types of ADEs in particular: hemorrhages and venous and arterial thromboembolisms, which will be defined according to international references [53-55].

Similarly, potential confounding factors in the causal relationship between antithrombotic drugs and related ADEs will be identified from scientific literature. They will include concomitant drugs (eg, drugs that modulate up or down the coagulation process or affect the metabolism or elimination of anticoagulants), patient characteristics (eg, congenital deficit in coagulation factors), and concomitant health conditions (eg, cirrhosis and kidney failure).

Finally, relevant ADEs and confounding factors will be selected using a modified Delphi method, which combines the summarized evidence with the collective judgment of an expert panel [56-58]. The panel will comprise geriatricians, pharmacologists, pharmacists, and internists from the research team. These experts will assess several statements on ADE characteristics during a 2-round process. These characteristics will comprise clinical significance, association with quality of care, and relevant confounding factors. The first round will consist of an individual remote rating with no interaction among the panelists. During the second round, the experts will meet and reach a consensus on ADE selection and relevant confounding factors. In each round, each panelist will receive an individualized document showing the distribution of all the experts’ ratings together with the panelist’s specific ratings.

**Direct and indirect adverse drug event (ADE) indicators.**

**ADE indicators**

- ADE markers: clinical signs or symptoms, diagnostic or treatment procedures, prescription and imaging orders, and biological test results indicating that an antithrombotic-related ADE occurred (ie, event)
- ADE triggers: diagnostic procedures, prescription and imaging orders, and biological test results that indicate that an antithrombotic-related ADE should have occurred but did not (ie, near miss)
- ADE confounding conditions and risk factors: conditions or factors that increase the risk of antithrombotic-related ADEs or the occurrence of a spontaneous bleeding or thrombotic event; risk factors include patient characteristics, specific concomitant health conditions that may interact with antithrombotic drugs, and concurrent use of more than one antithrombotic drug

### Data Extraction and Management (WP 2)

#### Overview

Structured data and free texts will be extracted from EMRs by the IT department of each hospital. Before being processed, structured data will be standardized in a unique common format (see the common data model in Tables S1-S4 in Multimedia Appendix 1), and unstructured data will be transformed into a machine-readable format when necessary.

Owing to the multisite and bilingual (ie, French and German) nature of the project, we will use a centralized and decentralized data governance strategy. A 2-step approach will be undertaken for data processing.

#### Step 1 (Decentralized Data Processing)

Raw data from the study participants’ EMRs will be managed and processed by the IT team of each hospital according to established protocols. Nominative identifiers of the structured data will be coded, and a table of correspondence between the original and coded identifiers will be stored on a protected server of each IT team. There are 18 identifiers to delete as described in the Health Insurance Portability and Accountability Act Privacy Rule, Code of Federal Regulations [59] Title 45: Public Welfare, Subtitle A §164.514. Free-text data of the learning set will be deidentified and coded locally by an encryption software before being transferred to secured servers dedicated to the project within each hospital.

#### Step 2 (Centralized Data Processing)

Locally extracted coded data of the learning set from the German-speaking part of Switzerland (USZ and KSB) and the French-speaking part (LUH and GUH) will be transferred to a centralized common SwissMADE database (DB). After proof of deidentification, structured items from free texts and narratives will be transferred to the SwissMADE DB. One authorized person per site will be allowed access to personal data and will decide who can access which data linked to which analysis. Remote access to the local working DBs within each hospital and the SwissMADE DB to authorized investigators will be made possible through a virtual private network.

### Data Analysis (WP 3 and 4)

Part of the structured and unstructured data will be used to develop ADE detection algorithms (working set), and another part will be used for validation (validation set). To test the algorithms’ accuracy, we will randomly select a validation data set to verify in the corresponding EMRs (gold standard) whether an ADE has truly occurred. As a result, the algorithms will be improved according to the results of the validation (ie, maximization of sensitivity and specificity as well as of positive predictive values [PPVs] and negative predictive values [NPVs]). Finally, validated algorithms will serve to identify ADEs accurately (ie, validated outcomes). For each 2015 and 2016 hospital stay of patients aged ≥65 years treated with antithrombotics, we will obtain a Case ID of positive ADE detected from the developed algorithms based on structured data (SDM), free-text data (NLP), and both types of data (SDM+NLP).

### Elaboration of Algorithms Based on Structured Data (WP 3)

Computational algorithms based on logical rules applied to structured data will be developed to identify ADE markers, triggers, confounding conditions or risk factors, and causes. Detection algorithms for clinical markers of ADEs (ie, hemorrhagic events or thromboembolism) and confounding clinical conditions (eg, chronic liver or kidney disease, hypertension, diabetes, cancer, and multimorbidity) will target ICD-10-German Modification diagnostic codes in hospital discharge data [60-62]. Regarding ADE triggers, clinical conditions (eg, hypotension, shock, and acute kidney failure) and procedures (eg, postoperative control of hemorrhage, drainage of hematoma, or surgical treatment of venous or arterial thromboembolism) will be identified from hospital discharge data using algorithms based on ICD-10-German Modification diagnostic codes and CHOP codes, respectively. Biological triggers of ADEs (ie, abnormal laboratory values) will be detected by algorithms applied to laboratory test results. Similarly, some algorithms based on prescription orders will search for pharmacological triggers of ADEs, including sudden medication stop orders, antidote ordering, dose correction orders, underdosing and overdosing, misprescribing, insufficient monitoring, and drug-drug or drug-disease associations.

In addition to the rule-based algorithms, data-driven algorithms will be created from the same data. Thus, we will perform predictive modeling of ADEs using penalized and nonpenalized logistic regression models with backward selection of predictors based on the Akaike information criterion and supervised and unsupervised machine learning approaches (eg, random forest, cluster analysis, and neural networks).

### Elaboration of Resources and Algorithms for Free-Text Data Processing (NLP; WP 4)

#### Overview

To develop models for classifying free-text discharge summaries as containing or not containing an ADE, multiple approaches will be considered. We divide these approaches into 2 categories: *pure NLP* approaches that require little to no medical or pharmaceutical expertise and hybrid approaches that are performed in close collaboration with medical and pharmaceutical experts. Both types of approaches require that we have access to a data set of discharge summaries that have been classified as containing or not containing an ADE. Pure NLP approaches include but are not limited to word and document embeddings, Bidirectional Encoder Representations from Transformers (BERT) for document classification, and other modern NLP methodologies. For hybrid approaches, we wish to build a named-entity recognition (NER) model that automatically annotates discharge summaries with various entities related to and relationships with ADEs. NER is a well-studied task in NLP in which a model learns to detect and label the mention of any predefined entity (ie, symptom, disease, and drug) in a span of unstructured text. Such a model will provide us with structured information from the free text that we could then exploit by designing rule-based algorithms or machine learning classification models using the entities as derived features. We note that more methods may be used to develop better classification models depending on the classification scores obtained using the aforementioned methods.

#### Pure NLP Approaches

These approaches require only a set of discharge summaries classified as containing or not containing an ADE. We can then, upon some preprocessing of the summaries, apply various NLP methods for classification. Popular methods include word and document embedding, BERT for document classification, and other modern approaches.

Word embeddings (respectively document embeddings) are a class of functions that transform a high-dimensionality space of words (respectively documents) into information-rich vectors in a lower-dimension embedding space, which can be used for evaluating the word-to-word (respectively document-to-document) similarity or used as a feature for more complex models. Recently, there have been promising results for several document classification tasks such as patient classification [63] or legal document classifications [64]. BERT [65] is a language model published in 2019 that can be used for a variety of tasks such as translation [66], question answering [67], and document classification. Although the original BERT model is based on the English language, French and German variants have since been published [68-70].

#### Hybrid Approaches

In this approach, we wish to leverage medical and pharmaceutical expertise to complement NLP methods. This hybrid approach can be seen as approximating the behavior of an expert classifying a discharge summary as “containing” or “not containing” an ADE. As a first step, we build an NER model to highlight various entities and connections that are highly related to ADEs in the discharge summaries. We focus on the following entities: antithrombotic drugs, other drugs, dosages, risk factors, hemorrhagic events, and thrombotic events. We also identify the relationships between a drug and its corresponding dosage as well as hemorrhagic and thrombotic events that happened in the past and not during the hospital stay. Already, domain expertise is required to build dictionaries of drugs and risk factors for the events we wish to detect. These sets of concepts will be organized in a coherent and pertinent taxonomy validated by pharmacologists, pharmacists, and geriatricians from the research team. To build an NER model, we rely on data annotated with the various entities that we want to predict. To work with gold-standard annotated data, we follow a robust annotation protocol inspired by Gurulingappa et al [71], which guarantees consistency between different annotators. Indeed, highly technical annotations such as those we are performing in our context can lead to disagreements between annotators. We mitigate this risk through several rounds of protocol harmonization as well as annotation review—each letter is independently annotated by 2 annotators and then reviewed by a third annotator. To build the NER model, we can rely, for instance, on the 2018 National NLP Clinical Challenges shared task [72]. This shared task focused on the detection of ADEs and related entities from clinical records. It led to breakthrough results (best overall *F*_1_-score of 0.94 on similar entities), and we intend to replicate some of the methods on our French and German corpora. We will also leverage new language models that have arisen since, such as BERT (and, more specifically, its French and German counterparts). With a data set of discharge summaries that have been classified as “containing” or “not containing” an ADE, we can then use features extracted with our NER model to train a machine learning classification model. We can also design rule-based algorithms based on domain expertise.

Eventually, NLP models for text annotation and ADE detection will be deployed as part of a pipeline that takes raw clinical text as input and outputs annotated text files with a list of detected ADEs, confounding conditions, and their probability scores.

### ADE Detection Tool Assessment (WP 5)

To assess the performance (eg, sensitivity, specificity, PPVs, and NPVs) of the ADE detection tool, a validation will be performed on a random sample of 600 hospital stays. ADE occurrence, type, causality, severity, and preventability will be assessed by means of a patient medical record review and analyzed by a team of pharmacologists, pharmacists, and geriatricians from the research team.

To ensure that the ADE assessment and data abstraction are structured and reliable, pharmacologists and pharmacists from the research team will develop a common ADE assessment form both in French and German based on existing good pharmacovigilance practice rules that will be disseminated in its original languages and English after study completion. Pairs of trained clinicians (eg, pharmacists, pharmacologists, and geriatricians) will then assess all selected medical records using this form. The causality between taking a drug and the advent of an ADE will be assessed using existing causality assessment scales [73,74]. The severity of the ADEs will be scored according to the Common Terminology Criteria for Adverse Events. Finally, an ADE will be deemed preventable if it was caused by a medication error that occurred during prescribing, transcribing, dispensing, administering, and monitoring or if it was due to a lack of medication adherence [75].

To test the reliability of the ADE assessment by pharmacists and pharmacologists, we will calculate intra- and interrater agreements for overall ADE occurrence, causality, severity, and preventability. For each pair of trained pharmacists and pharmacologists, the interrater agreement will be tested by comparing the results of the ADE assessment between members of the pair. To test for intrarater agreement in each participating hospital, a random sample including 100 ADEs detected by SDM and NLP will be reassessed by the assigned pair 3 months after their first assessment. For both intra- and interrater agreements, the measure of agreement will be the Cohen or uniform κ statistic [76]. After excluding false-positive ADEs, we will measure the cumulative incidence of true positive pADEs and nonpreventable ADEs for each hospital and medical unit as well as overall.

### Ethics Approval

The research project was approved by all cantonal ethics commissions involved in the project: Commission cantonale d'Ethique de la Recherche sur l'être humain Vaud" (CER-VD), Ethikkommission Nordwest- un Zentralscheiz (EKNZ), Commission cantonale d'éthique de la recherche Genève (CCER) and Kantonale Ethikkommission Zürich, under approval CER 2018-00272. Obtaining ethical permission is necessary for every study involving human participants. A method in which study participants can be satisfied that potential hazards have been evaluated, minimized, and declared acceptable is through the ethical review process.

## Results

### Health Data and Study Populations

After accounting for the inclusion and exclusion criteria, we will include 34,522 residents aged ≥65 years in our study (n=5888, 17.06% from LUH; n=11,581, 33.55% from GUH; n=8986, 26.03% from KSB; and n=8067, 23.37% from USZ).

### Sample Size Calculation for Geriatric Patient Safety Indicators Criterion Validity Assessment

The sample size was estimated to assess the performance of the SDM+NLP tool in detecting hemorrhagic adverse events. Indeed, the SDM+NLP tool is considered the critical outcome determining project feasibility, and hemorrhage is considered the most important adverse event related to antithrombotic drugs. We used a test result–based sampling method to minimize the number of medical records to be abstracted [77]. Given that CI is the cumulative incidence of ADEs detected from both structured and unstructured data, N is the number of 2016 hospital stays of patients at risk (ie, patients aged ≥65 years treated with antithrombotic drugs), p(ADE+) is the proportion of hospital stays with an ADE detected by the SDM+NLP tool among all at-risk hospital stays (calculated as the number of true positive and false-positive ADEs detected divided by N), Se is the expected sensitivity of the SDM+NLP tool, Sp is the expected specificity of the SDM+NLP tool, PPV, and NPV. We calculated the sample sizes for CI ranging from 3% to 24%, a desired Se of 80%, a 20% width for the 95% CI of Se, volumes of at-risk hospital stays ranging from N=2000 to N=20,000, and a balanced sample of ADE+ and ADE−hospital stays (ie, hospital stays with and without ADEs, respectively; Textbox 2). CI values were obtained from the literature (ie, a range of 30%-40% for ADE cumulative incidence and a range of 10%-40% for proportion of hemorrhagic ADEs among ADEs). The values of N were estimated from annual numbers of at-risk stays in the 4 participating hospitals. In particular, we considered selecting hospital units with a high prevalence of antithrombotic prescriptions (ie, acute geriatric unit, internal medicine, cardiology, angiology, orthopedic surgery, thoracic surgery, and cardiovascular surgery), which would increase the number of at-risk stays. The frequencies of 2015 at-risk stays in these selected units were 4711, 5130, 5564, and 5016 for LUH, GUH, USZ, and KSB, respectively (N=20,421). Therefore, we made the assumption that the number of at-risk stays for the 2015 to 2016 period would approximate 40,000. The sample size calculation was performed using Stata IC (version 14; StataCorp LLC). Thus, assuming CI equals 10% and p(ADE+) equals 12%, with 40,000 at-risk hospital stays and an expected Se equal to 80% with a 20% width for its 95% CI, a random sample of at least 523 medical records (51 ADE+ medical records and 472 ADE–medical records) will be necessary to assess the SDM+NLP tool. Considering that some medical records might not be available (perhaps 1%), the validation will finally require 530 medical records. Thus, we will abstract 15 medical records flagged as ADE+ and 120 medical records flagged as ADE–by the SDM+NLP tool during the 2015 to 2016 period in each of the 4 participating hospitals (135 medical records per hospital). Under these assumptions, the expected values and CIs for Se, Sp, PPV, and NPV should be as follows: Se=80% (95% CI 68%-88%), Sp=96% (95% CI 94%-97%), PPV=67% (95% CI 52%-79%), and NPV=98% (95% CI 96%-99%).

Regarding the external validation of predictive models, we will compare predicted outcomes with true outcomes based on medical record screenings for a small sample of 20 to 30 hospital stays for which predicted and validated outcomes diverge.

### Project Timetable

All structured and free-text data are now available to all research teams. However, the planned time frame for data extraction and the start of analyses was delayed by almost 1 year because of difficulties in obtaining approval from the 4 ethics committees. We also underestimated the challenges and time required to extract data from the 4 participating hospitals. Hospital information systems are not interoperable, so we had to provide additional resources to meet this challenge during 2021 and 2022. In addition, the barriers to data transfer or sharing from these hospitals despite ethics approval were highly unexpected. The data are being analyzed in 2022. We have completed ADE identification rules based on coded data (ICD-10 and CHOP codes) for all ADEs. Similar procedures will be applied to quantify the frequency of other ADEs related to antithrombotics and evaluate whether additional sources of information (laboratory values, drug prescriptions, and free text) will improve the detection algorithms. Tools for automatic annotation of free text (eg, exit letters) were developed in late 2021 and mid-2022 for the automated annotation of free texts (ie, discharge letters). They focus on drugs and symptoms for the German-language–based pipeline and on drugs, events, and risk factors for the French-language–based pipeline. These tools are in the validation phase in mid-2022, consisting of the “manual” revision and annotation of 600 (ZH) and 300 (CHUV) documents. Several machine learning models were trained and tested on a data set of 334 documents. Dictionaries specific to the medical languages encountered in these documents have been developed to explore the texts using both rule-based and machine learning algorithms. The research project will run until the end of 2022 to mid-2023.

## Discussion

### Overview

This interdisciplinary and integrative project involves 4 hospitals and experts with a background in different disciplines. Apart from the core competencies in clinical research and pharmacology, extracting meaningful information from electronic health records requires competencies in data analysis and NLP. First, we ensured data availability at each participating hospital. Moreover, summary statistics provided by each hospital show a good representativeness of the target population and of the medical or surgical units. The choice of antithrombotic drugs in the geriatric population should guarantee sufficient data to build the SDM and NLP tools. Although some feasibility risk exists with the NLP part of the project (ie, suboptimal specificity and sensitivity), the information gathered during the NLP process and all other project components (pharmacology and SDM) will provide new and relevant data on the safety of antithrombotics in the geriatric population.

This is the first study aiming at developing and validating a reliable automated tool to detect ADEs in older hospitalized patients using different data sources in Switzerland. Currently, only structured data are considered of value for the computerized patient record. However, the use of natural interfaces such as language analytics, which is being developed for the English language, has yet to expand to the German and French landscapes of health care systems. Assessing the quality and safety of antithrombotic therapy in older inpatients based on such data is innovative. This project will be able to leverage the importance of expressiveness in clinical free text and narratives by demonstrating that an analytical approach is applicable to such sources, thus fostering the possibility of using them for numerous other purposes such as within the Swiss Personalized Health Initiative.

The greater understanding of the development of ADEs as a result of antithrombotic therapies, including the identification of important contributing factors, the early recognition of repeating events, and the setting up of preventative measures for sustained risk reduction, will have a major impact on the increasing population of older patients in hospitals and at particular risk of toxicity. Although antithrombotic therapy is highly recommended and commonly prescribed in the older adult population [27], antiplatelet and anticoagulant treatments have been shown to be highly associated with bleeding complications [25-27], which are a major cause of emergency department admissions and mortality in this population [27,78]. Vulnerable older inpatients experience more ADEs compared with younger adults. These adverse events result in considerable morbidity and mortality and frequent institutionalization. They also considerably affect patients’ quality of life and their confidence in the health care system and health care professionals. Some ADEs cannot be prevented, but most are associated with poor patient safety and quality of care [2,16,23,79,80]. Moreover, older patients are underrepresented in trials, and accurate information regarding the benefit-risk balance of most drugs in this population is limited [22,23,27,29,35,36]. Classen et al [6] wrote that “identification and measurement of adverse medical events is central to patient safety, forming a foundation for accountability, prioritizing problems to work on, generating ideas for safer care, and testing which interventions work.” We also claim that detecting and monitoring ADEs in older inpatients, in particular pADEs, is the most important step to reducing age-related disparities in patient safety and, therefore, health inequities.

The technical and methodological aspects of the project (with multimodal, multisource, and multicentric data management) are the third strong point of the study. Owing to the multicomponent and multisite nature of the project, all organizational aspects to ensure effective scientific interactions between disciplines and hospitals throughout the research process will be disseminated for the Swiss research community. The algorithms used for SDM and NLP with all key information on data extraction and data mining will be made available (as open source) for further developments of medical NLP tools. Logical rules developed for SDM and the text-mining pipeline are electronic applications that can be directly implemented in hospital information systems. However, adapting automated detection tools to various hospital information systems and ADEs has proven to be difficult, but our ambitious and stepwise project will benefit from an interdisciplinary and experienced research team. We will focus on the 5 most clinically significant antithrombotic drug classes and plan to extend this project to other drug classes in the future. This tool could be implemented within EMRs and completed by e-alerts and reminders notifying providers of probable ADEs. It may also feature an automated causality assessment facilitating pharmacovigilance reports. This automated version of the Global Trigger Tool may help improve adverse drug effects and reaction reporting to local pharmacovigilance centers—and, therefore, to the Swiss authority for therapeutic products—while consuming fewer resources and being faster than the manual version currently in use.

Finally, our project is consistent with the Swiss Federal Council “Health 2030” agenda that advocates for “better data” to inform health policy and patients’ choice and improve quality [81], safety, and efficiency in vulnerable patients, particularly in older inpatients at high risk of developing ADEs. Although ADE reporting by health care professionals has regularly improved between 2002 and 2019, Swissmedic suspects considerable underreporting. Frequent and common adverse drug reactions (ADRs), even severe ones, are less subject to professionals’ focus than rare and new events [82]. Therefore, ADRs only partially reflect the real ADE prevalence. Presuming that the nationwide pharmacovigilance DB collects ADRs that are subject to selection and underreporting and given the lack of systematic ADE reporting and monitoring in Swiss hospitals, new strategies encompassing more comprehensive and systematic detection of ADEs are needed.

### Conclusions and Perspectives

This innovative study aiming to develop and validate an electronic application for the automated detection of ADEs related to antithrombotics will allow for the introduction of measures aimed at improving safety when prescribing antithrombotic medication. The increased performance of NLP as an important complement to structured data will bring existing tools to another level of efficiency in the detection of ADEs. Currently, such systems are not available in Switzerland, nor can “ready-made” systems from other countries be adapted as they are language-dependent. We hope that, in the near future, with these new types of tools developed for the French and German languages, the specificity of alerts will be improved, notifications will be prioritized, and clinical decision support will become more patient-centered.

## Appendix

Multimedia Appendix 1 Supplementary tables.

Multimedia Appendix 2 Evaluation of the protocol by the Swiss National Science Foundation.

Multimedia Appendix 3 Decision on the protocol submitted to the Swiss National Research Fund.

## Abbreviations

ADE: adverse drug event
ADR: adverse drug reaction
BERT: Bidirectional Encoder Representations from Transformers
CHOP: Swiss classification of surgical interventions
DB: database
EMR: electronic medical record
GUH: Geneva University Hospital
ICD-10: International Classification of Diseases, 10th Revision
KSB: Baden Cantonal Hospital
LUH: Lausanne University Hospital
NER: named-entity recognition
NLP: natural language processing
NPV: negative predictive value
pADE: preventable adverse drug event
PPV: positive predictive value
SDM: structured data mining
USZ: Zürich University Hospital
WP: work package
