# Supplementary material for: Automatic Detection of Adverse Drug Events in Geriatric Care: Study Proposal
Source: JMIR Res Protoc. 2022 Nov 15;11(11):e40456. doi: 10.2196/40456 (PMC9709671; doi:10.2196/40456)
Supplement: Multimedia Appendix 1 [file resprot_v11i11e40456_app1.docx]

**Multimedia Appendix 1**

**Automatic detection of adverse drug events in the geriatric care: study protocol_Supplement**

Manuscript entitled “Automatic detection of adverse drug events in the geriatric care: study protocol” by Gaspar Frédéric, et al

Appendices

Table S1 – Common data model for structured data (except laboratory variables)

Table S2 – Common data model for laboratory variables

Table S3 – Common data model for free text data

Table S4 – Justification of extracted items for structured data

Table S5 – ATC Codes of drugs concerned by the study

Table S1: Common data model for structured data (except laboratory variables)

| Variables of the **CDM** | | | | | |
| --- | --- | --- | --- | --- | --- |
| Category | Variable name | Description | Type | Format and size | String Specifications |
| General Administrative Data | PERSON_ID | Patient identification number | Factor | XXXXXXXXXXXXXXXX(16) |  |
| General Administrative Data | HOSPITALIZATION_ID | Case identification number (admission ID, hospitalization ID, or stay ID) | Factor | XXXXXXXXXXXXXXXX(16) |  |
| General Administrative Data | INSURANCE_TYPE | Coverage of basic health care: The main health insurance (third-party payer) that pays for the hospital stay in a general ward. | Factor | 1-9 (1) | 1=Mandatory statutoryhealth insurance  2=Disability Insurance  3=Military Insurance  4=Accident insurance  5=Self-payment (e.g.  foreigners without  insurance)  8=Other  9=Unknown |
| General Administrative Data | RESIDENCE_REGION | Region of residence (MedStat region) | Factor | AG01-ZH99(4) | Code ISO |
| General Administrative Data | NATIONALITY | Nationality | Factor | XX (2) | Code ISO |
| General Administrative Data | DATE_OF_BIRTH | Date of birth | Date | YYYY-MM-DD (8) |  |
| General Administrative Data | ADMISSION_AGE | Age at admission | Integer | 0-135 (3) |  |
| General Administrative Data | GENDER | Gender | Boolean | 1/2 (1) | 2= Female  1= Male |
| General Administrative Data | DATE_OF_DEATH | Date of death | Date | YYYY-MM-DD (8) |  |
| General Administrative Data | MEDICAL_CARE | Medical care | Factor | 1 = Ambulatory  3 = Hospitalization  9 = Unknown |  |
| Clinical measurements | BLOOD_PRESSURE_TAS | Blood pressure (TAS) | Integer | 1-999(3) |  |
| Clinical measurements | BLOOD_PRESSURE_TAD | Blood pressure (TAD) | Integer | 1-999(3) |  |
| Clinical measurements | CARDIAC_FREQ | Cardiac frequency | Integer | 1-999(3) |  |
| Clinical measurements | WEIGHT | Weight | Integer | 1-999 (3) |  |
| Clinical measurements | HEIGHT | Height | Integer | 1-999 (3) |  |
| Clinical measurements | ~~ALCOHOL_SCORE~~ | ~~Sum of alcohol withdrawal syndrome score~~ | String |  | **Variable not retained** |
| Clinical measurements | HEMATURIA | Hematuria | Boolean | +/- (1) | += Positive  - = Negative |
| Clinical measurements | BLOOD_IN_STOOL | Blood in Stool | Boolean | Positive/Negative (1) | Positive = Presence of blood in the stool  Negative = No blood in the stool |
| Patient location(s) and transfers | ADMISSION_DATE | Date of admission | Date | YYYY-MM-DD (8) |  |
| Patient location(s) and transfers | BEFORE_ADMISSION | Patient location before admission | Factor | 1-9 (1) | 1 = Home  2 = Home with care at  home  3 = Establishment of  non-hospital health  medicalized  4 = Establishment of  non-hospital health  unsafe  5 = Psychiatric Hospital  6 = Other hospital (care  acute) or house of  birth  7 = Implementing Institution  penalties  8 = Other  9 = Unknown |
| Patient location(s) and transfers | UNIT_HOSPITALIZATION | Unit of hospitalization | Factor | M000-M990 (4) | M000 = Medical Disciplines  M050 = Intensive care  M100 = Internal Medicine  M200 = Surgery  M300 = Gynecology and obstetrics  M400 = Pediatrics  M500 = Psychiatry and psychotherapy  M600 = Ophthalmology  M700 = Otorhinolaryngology  M800 = Dermatology and Venereology  M850 = Medical Radiology  M900 = Geriatrics  M950 = Physical Medicine and Rehabilitation  M960 = Emergency Centers  M970 = Physician Emergency Room  M990 = Other areas of activity |
| Patient location(s) and transfers | ADMISSION_MODE | Admission mode | Factor | 1-9 (1) | 1 = Emergency  2 = Announced, planned  3 = Birth (child born  Intramural)  4 = Internal transfer  5 = Transfer in the 24hours  8 = Other  9 = Unknown |
| Patient location(s) and transfers | HOSPITALIZATION_DECISION | Hospitalization decision | Factor | 1-6 / 8-9 (1) | 1 = Own initiative,  relatives  2 = Rescue service  (ambulance, police)  3 = Doctor  4 = Therapist not  doctor  5 = Social services  6 = Judicial authorities  8 = Other  9 = Unknown |
| Patient location(s) and transfers | TRANSFERS | Type of transfer | String | ? | 1= medicine  2= surgery  3= intermediate care  4= intensive care |
| Patient location(s) and transfers | DATE_TIME_OF_TRANSFERS | Date and time of transfers | Date and hour | YYYY-MM-DD-HH(10) |  |
| Patient location(s) and transfers | DATE_TIME_OF_ENTRY | Date and time of admission | Date and hour | YYYY-MM-DD-HH(10) |  |
| Patient location(s) and transfers | DATE_TIME_OF_EXIT | Date and time of exit | Date and hour | YYYY-MM-DD-HH(10) |  |
| Patient location(s) and transfers | EXIT_DECISION | Exit decision | Factor | 1-5 / 8-9 (1) | 1 = Initiative of the doctor  2 = Initiative of the patient  3 = Initiative of a third party  4 = Internal transfer  5 = Died  8 = Other  9 = Unknown |
| Patient location(s) and transfers | AFTER_EXIT | After exit | Factor | 0-5 / 8-9 (1) | 1 = Cured  2 = Care or treatment ambulatory  3 = Home care  4 = Care or treatment stationary  5 = Rehabilitation ambulatory or  stationary  8 = Other  9 = Unknown  0 = Deceased |
| Patient location(s) and transfers | READMISSION_1_ENTRY | 1st readmission entry date | Date | YYYY-MM-DD (8) |  |
| Patient location(s) and transfers | READMISSION_1_REASON | Reason for the 1st readmission | Factor | 1-9/empty (1) | Empty (= default)   1 = readmission with the   same MDC   2 = readmission with the   same MDC because   complications   3 = retransfer   9 = unknown |
| Patient location(s) and transfers | READMISSION_1_EXIT | 1st readmission exit date | Date | YYYY-MM-DD (8) |  |
| Patient location(s) and transfers | READMISSION_2_ENTRY | 2nd readmission entry date | Date | YYYY-MM-DD (8) |  |
| Patient location(s) and transfers | READMISSION_2_REASON | Reason for the 2nd readmission | Factor | 1-9/empty (1) | Empty (= default)   1 = readmission with the   same MDC   2 = readmission with the   same MDC because   complications   3 = retransfer   9 = unknown |
| Patient location(s) and transfers | READMISSION_2_EXIT | 2nd readmission exit date | Date | YYYY-MM-DD (8) |  |
| Patient location(s) and transfers | READMISSION_3_ENTRY | 3rd readmission entry date | Date | YYYY-MM-DD (8) |  |
| Patient location(s) and transfers | READMISSION_3_REASON | Reason for the 3rd re-admission | Factor | 1-9/empty (1) | Empty (= default)   1 = readmission with the   same MDC   2 = readmission with the   same MDC because   complications   3 = retransfer   9 = unknown |
| Patient location(s) and transfers | READMISSION_3_EXIT | 3rd readmission exit date | Date | YYYY-MM-DD (8) |  |
| Patient location(s) and transfers | READMISSION_4_ENTRY | 4th readmission entry date | Date | YYYY-MM-DD (8) |  |
| Patient location(s) and transfers | READMISSION_3_REASON | Reason for the 4th re-admission | Factor | 1-9/empty (1) | Empty (= default)   1 = readmission with the   same MDC   2 = readmission with the   same MDC because   complications   3 = retransfer   9 = unknown |
| Patient location(s) and transfers | SUBSEQUENT_READMISSIONS | Further readmissions | Factor | 0/1 (1) | 0 = (default), not more than 5   stays (i.e., 4   readmissions)   1 = more than 5 stays (4   readmissions) |
| Patient location(s) and transfers | READMISSION_4_ENTRY | 4th readmission entry date | Date | YYYY-MM-DD (8) |  |
| Patient location(s) and transfers | READMISSION_3_REASON | Reason for the 4th re-admission | Factor | 1-9/empty (1) | Empty (= default)   1 = readmission with the   same MDC   2 = readmission with the   same MDC because   complications   3 = retransfer   9 = unknown |
| Patient location(s) and transfers | SUBSEQUENT_READMISSIONS | Further readmissions | Factor | 0/1 (1) | 0 = (default), not more than 5   stays (i.e., 4   readmissions)   1 = more than 5 stays (4   readmissions) |
| Diagnoses and procedures | DRG | Swiss Diagnosis Related  Groups | Factor | ? | DRGs codes |
| Diagnoses and procedures | MAIN_DIAGNOSIS | Main diagnosis | String | A00000-Z9999(6) | ICD-10-GM |
| Diagnoses and procedures | MAIN_ DIAGNOSIS_LAT | Laterality for the main diagnosis | Factor | 0 = bilateral  1 = unilateral right  2 = unilateral left  3 = one-sided unknown side  9 = unknown  Empty = the question does not arise | 0-3/9/vide (1) |
| Diagnoses and procedures | MAIN_DIAGNOSIS_TUMOR | Diagnosis tumor activity | Factor | 0 = inactive  1 = active  9 = unknown  empty = the question does not arise | 0/1/9/vide (1) |
| Diagnoses and procedures | SUPPLEMENT_DIAGNOSIS | Diagnostic Supplement | String | A00000-Z9999(6) | ICD-10-GM |
| Diagnoses and procedures | 1ADDITIONALD_LAT | Laterality of the 1st additional diagnosis | Factor | 0 = bilateral  1 = unilateral right  2 = unilateral left  3 = one-sided unknown side  9 = unknown  Empty = the question does not arise | 0-3/9/vide (1) |
| Diagnoses and procedures | 1ADDITIONALD_TUMOR | Tumor activity for the 1st diagnosis  additional | Factor | 0 = inactive  1 = active  9 = unknown  empty = the question does not arise | 0/1/9/vide (1) |
| Diagnoses and procedures | 2ADDITIONAL_DIAGNOSIS | 2nd additional diagnosis | String | A00000-Z9999(6) | ICD-10-GM |
| Diagnoses and procedures | 2ADDITIONALD_LAT | Laterality of the 2nd diagnosis  additional | Factor | 0 = bilateral  1 = unilateral right  2 = unilateral left  3 = one-sided unknown side  9 = unknown  Empty = the question does not arise | 0-3/9/vide (1) |
| Diagnoses and procedures | 2ADDITIONALD_TUMOR | Tumor activity for the 2nd  additional diagnosis | Factor | 0 = inactive  1 = active  9 = unknown  empty = the question does not arise | 0/1/9/vide (1) |
| Diagnoses and procedures | 3ADDITIONAL_DIAGNOSIS | 3rd additional diagnosis | String | A00000-Z9999(6) | ICD-10-GM |
| Diagnoses and procedures | 3ADDITIONALD_LAT | Laterality of the 3rd diagnosis  additional | Factor | 0 = bilateral  1 = unilateral right  2 = unilateral left  3 = one-sided unknown side  9 = unknown  Empty = the question does not arise | 0-3/9/vide (1) |
| Diagnoses and procedures | 3ADDITIONALD_TUMOR | Tumor activity for the 3rd  additional diagnosis | Factor | 0 = inactive  1 = active  9 = unknown  empty = the question does not arise | 0/1/9/vide (1) |
| Diagnoses and procedures | 4ADDITIONAL_DIAGNOSIS | 4th additional diagnosis | String | A00000-Z9999(6) | ICD-10-GM |
| Diagnoses and procedures | 4ADDITIONALD_LAT | Laterality of the 4th diagnosis  additional | Factor | 0 = bilateral  1 = unilateral right  2 = unilateral left  3 = one-sided unknown side  9 = unknown  Empty = the question does not arise | 0-3/9/vide (1) |
| Diagnoses and procedures | 4ADDITIONALD_TUMOR | Tumor activity for the 4th  additional diagnosis | Factor | 0 = inactive  1 = active  9 = unknown  empty = the question does not arise | 0/1/9/vide (1) |
| Diagnoses and procedures | 5ADDITIONAL_DIAGNOSIS | 5th additional diagnosis | String | A00000-Z9999(6) | ICD-10-GM |
| Diagnoses and procedures | 5ADDITIONALD_LAT | Laterality of the 5th diagnosis  additional | Factor | 0 = bilateral  1 = unilateral right  2 = unilateral left  3 = one-sided unknown side  9 = unknown  Empty = the question does not arise | 0-3/9/vide (1) |
| Diagnoses and procedures | 5ADDITIONALD_TUMOR | Tumor activity for the 5th  additional diagnosis | Factor | 0 = inactive  1 = active  9 = unknown  empty = the question does not arise | 0/1/9/empty (1) |
| Diagnoses and procedures | 6ADDITIONAL_DIAGNOSIS | 6th additional diagnosis | String | A00000-Z9999(6) | ICD-10-GM |
| Diagnoses and procedures | 6ADDITIONALD_LAT | Laterality of the 6th diagnosis  additional | Factor | 0 = bilateral  1 = unilateral right  2 = unilateral left  3 = one-sided unknown side  9 = unknown  Empty = the question does not arise | 0-3/9/empty (1) |
| Diagnoses and procedures | 6ADDITIONALD_TUMOR | Tumor activity for the 6th  additional diagnosis | Factor | 0 = inactive  1 = active  9 = unknown  empty = the question does not arise | 0/1/9/empty (1) |
| Diagnoses and procedures | 7ADDITIONAL_DIAGNOSIS | 7th additional diagnosis | String | A00000-Z9999(6) | ICD-10-GM |
| Diagnoses and procedures | 7ADDITIONALD_LAT | Laterality of the 7th diagnosis  additional | Factor | 0 = bilateral  1 = unilateral right  2 = unilateral left  3 = one-sided unknown side  9 = unknown  Empty = the question does not arise | 0-3/9/ empty (1) |
| Diagnoses and procedures | 7ADDITIONALD_TUMOR | Tumor activity for the 7th  additional diagnosis | Factor | 0 = inactive  1 = active  9 = unknown  empty = the question does not arise | 0/1/9/ empty (1) |
| Diagnoses and procedures | 8ADDITIONAL_DIAGNOSIS | 8th additional diagnosis | String | A00000-Z9999(6) | ICD-10-GM |
| Diagnoses and procedures | 8ADDITIONALD_LAT | Laterality of the 8th diagnosis  additional | Factor | 0 = bilateral  1 = unilateral right  2 = unilateral left  3 = one-sided unknown side  9 = unknown  Empty = the question does not arise | 0-3/9/ empty (1) |
| Diagnoses and procedures | 8ADDITIONALD_TUMOR | Tumor activity for the 8th  additional diagnosis | Factor | 0 = inactive  1 = active  9 = unknown  empty = the question does not arise | 0/1/9/ empty (1) |
| Diagnoses and procedures | 9ADDITIONAL_DIAGNOSIS | 9th additional diagnosis | String | A00000-Z9999(6) | ICD-10-GM |
| Diagnoses and procedures | 9ADDITIONALD_LAT | Laterality of the 9th diagnosis  additional | Factor | 0 = bilateral  1 = unilateral right  2 = unilateral left  3 = one-sided unknown side  9 = unknown  Empty = the question does not arise | 0-3/9/ empty (1) |
| Diagnoses and procedures | 9ADDITIONALD_TUMOR | Tumor activity for the 9th  additional diagnosis | Factor | 0 = inactive  1 = active  9 = unknown  empty = the question does not arise | 0/1/9/ empty (1) |
| Diagnoses and procedures | **…** | **…** | **…** | **…** | **…** |
| Diagnoses and procedures | 49ADDITIONAL_DIAGNOSIS | 49th additional diagnosis | String | A00000-Z9999(6) | ICD-10-GM |
| Diagnoses and procedures | 49ADDITIONALD_LAT | Laterality of the 49th diagnosis  additional | Factor | 0 = bilateral  1 = unilateral right  2 = unilateral left  3 = one-sided unknown side  9 = unknown  Empty = the question does not arise | 0-3/9/ empty (1) |
| Diagnoses and procedures | 49ADDITIONALD_TUMOR | Tumor activity for the 49th  additional diagnosis | Factor | 0 = inactive  1 = active  9 = unknown  empty = the question does not arise | 0/1/9/ empty (1) |
| Diagnoses and procedures | MAIN_INTERVENTION | Main Intervention | String | XXXXXX (6) | CHOP codes |
| Diagnoses and procedures | MAIN_ INTERVENTION_LAT | Laterality for main Intervention | Factor | 0-3 / 9 / vide (1) | 0 = bilateral  1 = unilateral right  2 = unilateral left  3 = one-sided unknown side  9 = unknown  Empty = the question does not arise |
| Diagnoses and procedures | MAIN_INTERVENTION_DATE | Start of main Intervention | Date | YYYY-MM-DD-HH(10) |  |
| Diagnoses and procedures | 1ADDITIONALI_INTERVENTION | 1st additional Intervention | String | XXXXXX (6) | CHOP codes |
| Diagnoses and procedures | 1ADDITIONALI_LAT | Laterality 1st line. additional | Factor | 0-3 / 9 / vide (1) | 0 = bilateral  1 = unilateral right  2 = unilateral left  3 = one-sided unknown side  9 = unknown  Empty = the question does not arise  not |
| Diagnoses and procedures | 1ADDITIONALI_DATE | Start of the 1st line. additional | Date | YYYY-MM-DD-HH(10) |  |
| Diagnoses and procedures | 2ADDITIONALI_INTERVENTION | 2nd additional Intervention | String | XXXXXX (6) | CHOP codes |
| Diagnoses and procedures | 2ADDITIONALI_LAT | Laterality 2nd line. additional | Factor | 0-3 / 9 / vide (1) | 0 = bilateral  1 = unilateral right  2 = unilateral left  3 = one-sided unknown side  9 = unknown  Empty = the question does not arise  not |
| Diagnoses and procedures | 2ADDITIONALI_DATE | Start of the 2nd line. additional | Date | YYYY-MM-DD-HH(10) |  |
| Diagnoses and procedures | 3ADDITIONALI_INTERVENTION | 3rd additional Intervention | String | XXXXXX (6) | CHOP codes |
| Diagnoses and procedures | 3ADDITIONALI_LAT | Laterality 3rd line. additional | Factor | 0-3 / 9 / vide (1) | 0 = bilateral  1 = unilateral right  2 = unilateral left  3 = one-sided unknown side  9 = unknown  Empty = the question does not arise  not |
| Diagnoses and procedures | 3ADDITIONALI_DATE | Start of the 3rd line. additional | Date | YYYY-MM-DD-HH(10) |  |
| Diagnoses and procedures | 4ADDITIONALI_INTERVENTION | 4th additional Intervention | String | XXXXXX (6) | CHOP codes |
| Diagnoses and procedures | 4ADDITIONALI_LAT | Laterality 4th line. additional | Factor | 0-3 / 9 / vide (1) | 0 = bilateral  1 = unilateral right  2 = unilateral left  3 = one-sided unknown side  9 = unknown  Empty = the question does not arise  not |
| Diagnoses and procedures | 4ADDITIONALI_DATE | Start of the 4th line. additional | Date | YYYY-MM-DD-HH(10) |  |
| Diagnoses and procedures | 5ADDITIONALI_INTERVENTION | 5th additional Intervention | String | XXXXXX (6) | CHOP codes |
| Diagnoses and procedures | 5ADDITIONALI_LAT | Laterality 5th line. additional | Factor | 0-3 / 9 / vide (1) | 0 = bilateral  1 = unilateral right  2 = unilateral left  3 = one-sided unknown side  9 = unknown  Empty = the question does not arise  not |
| Diagnoses and procedures | 5ADDITIONALI_DATE | Start of the 5th line. additional | Date | YYYY-MM-DD-HH(10) |  |
| Diagnoses and procedures | 6ADDITIONALI_INTERVENTION | 6th additional Intervention | String | XXXXXX (6) | CHOP codes |
| Diagnoses and procedures | 6ADDITIONALI_LAT | Laterality 6th line. additional | Factor | 0-3 / 9 / vide (1) | 0 = bilateral  1 = unilateral right  2 = unilateral left  3 = one-sided unknown side  9 = unknown  Empty = the question does not arise  not |
| Diagnoses and procedures | 6ADDITIONALI_DATE | Start of the 6th line. Additional | Date | YYYY-MM-DD-HH(10) |  |
| Diagnoses and procedures | 7ADDITIONALI_INTERVENTION | 7th additional Intervention | String | XXXXXX (6) | CHOP codes |
| Diagnoses and procedures | 7ADDITIONALI_LAT | Laterality 7th line. additional | Factor | 0-3 / 9 / vide (1) | 0 = bilateral  1 = unilateral right  2 = unilateral left  3 = one-sided unknown side  9 = unknown  Empty = the question does not arise  not |
| Diagnoses and procedures | 7ADDITIONALI_DATE | Start of the 7th line. additional | Date | YYYY-MM-DD-HH(10) |  |
| Diagnoses and procedures | 8ADDITIONALI_ INTERVENTION | 8th additional Intervention | String | XXXXXX (6) | CHOP codes |
| Diagnoses and procedures | 8ADDITIONALI_LAT | Laterality 8th line. additional | Factor | 0-3 / 9 / vide (1) | 0 = bilateral  1 = unilateral right  2 = unilateral left  3 = one-sided unknown side  9 = unknown  Empty = the question does not arise  not |
| Diagnoses and procedures | 8ADDITIONALI_DATE | Start of the 8th line. Additional | Date | YYYY-MM-DD-HH(10) |  |
| Diagnoses and procedures | 9ADDITIONALI_ INTERVENTION | 9th additional Intervention | String | XXXXXX (6) | CHOP codes |
| Diagnoses and procedures | 9ADDITIONALI_LAT | Laterality 9th line. additional | Factor | 0-3 / 9 / vide (1) | 0 = bilateral  1 = unilateral right  2 = unilateral left  3 = one-sided unknown side  9 = unknown  Empty = the question does not arise  not |
| Diagnoses and procedures | 9ADDITIONALI_DATE | Start of the 9th line. additional | Date | YYYY-MM-DD-HH(10) |  |
| Diagnoses and procedures | 10ADDITIONALI_ INTERVENTION | 10th additional Intervention | String | XXXXXX (6) | CHOP codes |
| Diagnoses and procedures | 10ADDITIONALI_LAT | Laterality 10th line. additional | Factor | 0-3 / 9 / vide (1) | 0 = bilateral  1 = unilateral right  2 = unilateral left  3 = one-sided unknown side  9 = unknown  Empty = the question does not arise  not |
| Diagnoses and procedures | 10ADDITIONALI_DATE | Start of the 10th line. additional | Date | YYYY-MM-DD-HH(10) |  |
| Diagnoses and procedures | **…** | **…** | **…** | **…** | **…** |
| Diagnoses and procedures | 99ADDITIONALI_ INTERVENTION | 99th additional Intervention | String | XXXXXX (6) | CHOP codes |
| Diagnoses and procedures | 99ADDITIONALI_LAT | Laterality 99th trait. additional | Factor | 0-3 / 9 / vide (1) | 0 = bilateral  1 = unilateral right  2 = unilateral left  3 = one-sided unknown side  9 = unknown  Empty = the question does not arise  not |
| Diagnoses and procedures | 99ADDITIONALI_DATE | Start of the 99th feature additional | Date | YYYY-MM-DD-HH(10) |  |
| Diagnoses and procedures | INTENSIVE_CARE_TIME | Time (hours) spent in the Intensive Care Unit(ICU) | Integer | 0-99999 (5) |  |
| Diagnoses and procedures | DURATION_OF_MECHANICAL_VENTILATION | Duration of mechanical ventilation (hours) | Integer | 0-99999 (5) |  |
| Diagnoses and procedures | DISEASE_SEVERITY | Disease severity (for stays in ICU only) | Integer | 0-999 (3) | SSMI score (SAPS, PIM2 or CRIB2) |
| Diagnoses and procedures | DISEASE_SCORE | Type of disease severity score used (for stays in ICU only) | Factor | S/P/C(1) | S = SAPS  P = PIM2  C = CRIB |
| Diagnoses and procedures | NEMS_SCORE | Workload score (nine equivalent of nursing manpower use score, ) NEMS), | Integer | 0-999999(6) | NEMS Score |
| Laboratory values |  |  |  |  |  |
| Prescription/Medication | MEDICATION_ATC | ATC code | String | XXXXXXX (7) | The Anatomical Therapeutic Chemical (ATC) Classification System |
| Prescription/Medication | MEDICATION_PRODUCTNAME | Product Name | String |  |  |
| Prescription/Medication | MEDICATION_GENERICNAME | Generic Product Name | String |  |  |
| Prescription/Medication | MEDICATION_DOSE | Product dose | Integer | 0-99999 (5) |  |
| Prescription/Medication | MEDICATION_UNIT | Administrated Unit | String |  |  |
| Prescription/Medication | MEDICATION_DATE_PRES | Date of the drug prescribed | Date | YYYY-MM-DD (8) |  |
| Prescription/Medication | MEDICATION_DATE_ADMIN | Date of the drug administered | Date | YYYY-MM-DD (8) |  |
| Prescription/Medication | MEDICATION_ACTIVESUB | Active substance | String |  |  |
| Prescription/Medication | MEDICATION_DATE_START | Prescription Start Date | Date | YYYY-MM-DD (8) |  |
| Prescription/Medication | MEDICATION_DATE_END | Prescription End Date | Date | YYYY-MM-DD (8) |  |
| Prescription/Medication | MEDICATION_FIRST_ADMIN | Date of first administration | Date | YYYY-MM-DD (8) |  |
| Prescription/Medication | MEDICATION_LAST_ADMIN | Date of Last Administration | Date | YYYY-MM-DD (8) |  |
| Prescription/Medication | MEDICATION_ADMIN_DOSE | Administered dose | Integer | 0-99999 (5) |  |
| Prescription/Medication | MEDICATION_ADMIN _VOLUME | Administrated volume | Integer | 0-99999 (5) |  |
| Prescription/Medication | MEDICATION _VOLUME_UNIT | Volume Unit | String |  |  |
| Prescription/Medication | MEDICATION_ADMIN_RATE | ~~Administrated debit~~ | Integer | 0-99999 (5) |  |
| Prescription/Medication | MEDICATION_RATE_UNIT | ~~Debit Unit~~ | String |  |  |
| Prescription/Medication | MEDICATION_DURATION | Administrated duration | Integer | 0-99999 (5) |  |
| Prescription/Medication | MEDICATION_ADMIN_FREQU | Administrated frequency | Integer | 0-99999 (5) |  |
| Prescription/Medication | MEDICATION_ROUTE | Route of administration | String |  |  |

Table S2 : Common data model for laboratory variables

| Variables of the CDM | | | | | |
| --- | --- | --- | --- | --- | --- |
| Category | Variable name | Description | Format/ Unit | Matrix | Reference interval |
| Electrolytes and ions | LAB_VALUE_NA | Sodium | Integer/ mmol/l | Serum | 135-150 |
| Electrolytes and ions | LAB_VALUE_POTASSIUM | Potassium | Integer/ mmol/l | Serum | 3.6-5 |
| Electrolytes and ions | LAB_VALUE_SERUM_LACTATE | Serum lactate | Integer/ mmol/l | Serum | 0.63-2.44 |
| Electrolytes and ions | LAB_VALUE_BICARBONATE | Bicarbonate | Integer/ mmol/l | Serum | 21.0-28.5 |
| Electrolytes and ions | LAB_VALUE_UREA | Urea | Integer/ mmol/l | Serum | 2.5-7.6 |
| Electrolytes and ions | LAB_VALUE_IRON | Serum iron | Integer/ µmol/l | Serum | F 9-28  M10-30 |
| Electrolytes and ions | LAB_VALUE_TRANSFERRIN | Serum Transferrin | Integer/ µmol/l | Serum | 24.7 - 44.4 |
| Electrolytes and ions | LAB_VALUE_TRANSFERRIN_SATUTATION | Transferrin saturation | Integer/ µmol/l | Serum | 45-72 |
| Electrolytes and ions | LAB_VALUE_SEUM_FERRITIN | Serum Ferritin | Integer/ μg/l | Serum | 24.7 - 44.4 |
| Enzymes | LAB_VALUE_AST | AST | Integer/ U/I | Serum | 7-40 |
| Enzymes | LAB_VALUE_ALT | ALT | Integer/ U/I | Serum | 5-35 |
| Enzymes | LAB_VALUE_CREATINE_KINASE | Creatine kinase (CK) | Integer/ U/I | Serum | F 20-160  M 20-215 |
| Enzymes | LAB_VALUE_GGT | Gamma-glutamyltransferase (GGT) | Integer/ U/I | Serum | F 12-43  M 15-73 |
| Enzymes | LAB_VALUE_ALP | Alkaline phosphatase (ALP) | Integer/ U/I | Serum | 40-160 |
| Complete Blood count (CBC) | LAB_VALUE_ERY | Red blood cell count | Integer/ T/l | Plasma | 4.4-5.8 |
| Complete Blood count (CBC) | LAB_VALUE_HB | Hemoglobin | Integer/ g/l | Plasma | F 117-157  M 133-177 |
| Complete Blood count (CBC) | LAB_VALUE_HT | Hematocrit | Integer/ % | Plasma | F 35-47  M 40-52 |
| Complete Blood count (CBC) | LAB_VALUE_LEUCO | White blood cell count | Integer/ G/l | Plasma | 4-10 |
| Complete Blood count (CBC) | LAB_VALUE_PLAQ | Platelet count | Integer/ G/l | Plasma | 150-350 |
| Hemostasis | LAB_VALUE_PT | Prothrombin time (PT) | Integer/ % | Sang total | 80-120 |
| Hemostasis | LAB_VALUE_APTT | Activated partial thromboplastin time (APTT) | Integer/ sec | Sang total | 26-37 |
| Hemostasis | LAB_VALUE_TT | Thrombin time (TT) | Integer/ sec | Sang total | 14-19 |
| Hemostasis | LAB_VALUE_INR | International normalized ratio (INR) | Integer | Sang total |  |
| Hemostasis | LAB_VALUE_FI | Plasma Fibrinogen | Integer/ g/l | Plasma | 2.0-4.0 |
| Hemostasis | LAB_VALUE_ANITHROMBIN | Antithrombin | Integer/ % | Plasma | 80-130 |
| Hemostasis | LAB_VALUE_PROTEIN_C | Protein C | Integer/ % | Plasma | 70-150 |
| Hemostasis | LAB_VALUE_PROTEIN_S | Protein S | Integer/ % | Serum | 70 |
| Hemostasis | LAB_VALUE_ANTICARDIOLIPIN_IGG | Anti-cardiolipin antibody IgG | Integer | Serum | 0-23 |
| Hemostasis | LAB_VALUE_ANTICARDIOLIPIN_IGM | Anti-cardiolipin antibody IgM | Integer | Serum | 0-11 |
| Hemostasis | LAB_VALUE_ANTIBETA | Anti-bêta-2-glycoprotéine 1 antibody | Integer/ U/ml | Serum | <7 : Negative  7-10 : Equivaocal  >10 : Positive |
| Hemostasis | LAB_VALUE_PROTHROMBIN_F1F2 | Fragments of prothrombin (F1+F2) | Integer/ nmol/l | Plasma | 0.1 |
| Hemostasis | LAB_VALUE_TAT | Thrombin–antithrombin complex (TAT) | Integer/ ug/l | Plasma | 1.0-4.1 |
| Hemostasis | LAB_VALUE_FACTOR_II | Coagulation Factor II | Integer/ % | Plasma | 50-150 |
| Hemostasis | LAB_VALUE_FACTOR_V | Coagulation Factor V | Integer/ % | Plasma | 50-150 |
| Hemostasis | LAB_VALUE_FACTOR_VII | Coagulation Factor VII | Integer/ % | Plasma | 50-150 |
| Hemostasis | LAB_VALUE_FACTOR_VIII | Coagulation Factor VIII | Integer/ % | Plasma | 60-130 |
| Hemostasis | LAB_VALUE_FACTOR_X | Coagulation Factor X | Integer/ % | Plasma | 50-150 |
| Hemostasis | LAB_VALUE_FACTOR_XI | Coagulation Factor XI | Integer/ % | Plasma | 50-180 |
| Hemostasis | LAB_VALUE_FACTOR_XII | Coagulation Factor XII | Integer/ % | Plasma | 40-150 |
| Hemostasis | LAB_VALUE_FACTOR_XIII | Coagulation Facotr XIII | Integer/ % | Plasma | 70-130 |
| Hemostasis | LAB_VALUE_DDIMER | D-Dimer | Integer/ ng/ml | Plasma | <500 |
| Hemostasis | LAB_VALUE_ANTIXA_ACTIVITY | Anti-Xa | Integer | ? |  |
| Hemostasis | LAB_VALUE_ANTIIIA_ACTIVTIY | Anti-IIa | Integer | ? |  |
| Other | LAB_VALUE_ALBUMIN | Albumin | Integer/ g/l | Serum | 35 - 52 |
| Other | LAB_VALUE_TOTAL_PROTEIN | Serum total protein | Integer/ g/l | Serum | 63-82 |
| Other | LAB_VALUE_OXYGEN_SATUATION | Oxygen saturation | Integer/ % |  | 95-100 |
| Other | LAB_VALUE_CRP | C-reactive protein (CRP) | Integer/ mg/l | Serum | < 5 |
| Other | LAB_VALUE_MB | Myoglobin (Mb) | Integer/ U/l | Serum | < 6% |
| Other | LAB_VALUE_TROPONIN | Troponin T | Integer | Plasma | <50: IDM peu probable mais possible 50-100:IDM possible 100-2000:IDM probable >2000: IDM très probable |
| Other | LAB_VALUE_CREATININE | Creatinine | Integer/ µmol/l | Urine | F 50 -100  M 65-120 |
| Other | LAB_VALUE_BILT | Total bilirubin | Integer/ μmol/l | Serum | 5-17 |
| Other | LAB_VALUE_BILD | Direct bilirubin | Integer/ μmol/l | Serum | 2-5 |
| Other | LAB_VALUE_GLYCHEMO | Glycated hemoglobin | Integer/ % | Serum | 4.3 - 5.7 |
| Other | LAB_VALUE_GFR | Glomerular filtration rate | Integer/ ml/min/1.73m2 |  | CKD stage GFR level  Stage 1 ≥ 90  Stage 2 60–89  Stage 3 30–59  Stage 4 15–29  Stage 5 < 15 |
| Other | LAB_VALUE_HEMATURIA | Hematuria | Integer/ | Urine |  |
| Laboratory values | For each laboratory variable, the following information is linked | Date of sampling of the test | Date |  |  |
| Laboratory values | For each laboratory variable, the following information is linked | Date of receipt of the test | Date |  |  |
| Laboratory values | For each laboratory variable, the following information is linked | Test analysis date | Date |  |  |
| Laboratory values | For each laboratory variable, the following information is linked | Test ID | Integer |  |  |
| Laboratory values | For each laboratory variable, the following information is linked | Type of analysis | String |  |  |
| Laboratory values | For each laboratory variable, the following information is linked | Test result | Integer/Text |  |  |
| Laboratory values | For each laboratory variable, the following information is linked | Unit of the test | Text |  |  |
| Laboratory values | For each laboratory variable, the following information is linked | Test comments | Text |  |  |

Table S3 : Common data model for free text data

| Category | Document name | Document description in proposal | Description | **Format** |
| --- | --- | --- | --- | --- |
| Free-text | Admission | Notes taken at admission | A document written by a clinician containing information taken during the admission of the patient and the goal of the stay | **.txt** |
| Free-text | Discharge | Discharge summaries and letters | A document written by a clinician summarizing the stay of the patient. Can be of mainly three types : discharge letter, transfer letter or death letter | **.txt** |
| Free-text | Nurse Progress Note | Nurses’ progress notes | Notes taken by nurses during the stay | **.txt** |
| Free-text | Radiology Report | Imaging/radiology reports | Any report coming from the radiology division and describing a radiologic exam of the patient | **.txt** |
| Free-text | Specialist Consultation | Specialists’ (e.g. hematologist, cardiologist, angiologist, and particularly endoscopy reports) consultation notes | A document summarizing the intervention of a specialist during the stay of the patient | **.txt** |
| Free-text | Pharmacology Consultation | Clinical pharmacology or pharmacy service consultation notes | A document summarizing the intervention of a pharmacologist during the stay of the patient (already included in Specialist consultation | **.txt** |
| Free-text | Pharmacovigilance Report | ADE / Pharmacovigilance Reports | Pharmacovigilance reports written during the stay of the patient | **.txt** |
| Free-text | CIRS Report | CIRS Reports | Report from the CIRS | **.txt** |

Table S4: Justification of extracted items for structured data

| Data type | (Descriptive) Justification | References |
| --- | --- | --- |
| General Administrative Data | Descriptive value | |
| Clinical measure |  |  |
| Blood pressure | (To identify blood pressure disorders)  Risk factor that influence hemorrhagic, thromboembolic events | (Zhu, He et al. 2015) |
| Weight/Height | Descriptive value and risk factor that influence hemorrhagic, thromboembolic events | (Lip, Frison et al. 2011, Bene, Dubart et al. 2014) |
| Sum of alcohol withdrawal syndrome score | Risk factor that influence hemorrhagic, thromboembolic events | (White, Beyth et al. 1999, Olesen, Lip et al. 2011) |
| Patient location(s) and transfers | Identify triggers that indicate that an antithrombotic-related ADE may have occurred |  |
| Diagnoses and procedures | (Select hospital stays eligible for the denominator of indicators)  Identify an hemorrhagic, thromboembolic event (markers) | (LI 2013, Le Pogam, Quantin et al. 2017, (AHRQ) 2017 ) |
| Laboratory values |  |  |
| Measured electrolytes and ions |  |  |
| Blood Ionogram: Sodium and Potassium | (Abnormal values ​​can be a sign of a vital organ failure)  Risk factor that influence hemorrhagic, thromboembolic events | (Kim, Ozonoff et al. 2015) |
| Lactic acid | (Abnormal values ​​may indicate different states of shock, severe anemia or ventricular failure)  Risk factor that influence hemorrhagic, thromboembolic events | (Ruiz-Gimenez, Suarez et al. 2008) |
| Uric acid | (To diagnose kidney disorder or to reveal hematopathies, chronic renal failure, severe hepatic insufficiency (with decreased uric acid synthesis) or signs of tumors)  Risk factor that influence hemorrhagic, thromboembolic events | (Beyth, Quinn et al. 1998) |
| Urea | (To assess kidney function and in particular to detect renal failure. High values can also be indicative of heart damage or gastrointestinal hemorrhage)  Risk factor that influence hemorrhagic, thromboembolic events | (Hirsh, Guyatt et al. 2008, Holbrook, Schulman et al. 2012) |
| Iron and Ferritin Level | (The determination of iron and ferritin makes the identification of anemia possible)  Identify an hemorrhagic, thromboembolic event (markers) and risk factor | (Ruiz-Gimenez, Suarez et al. 2008) |
| Measured enzymes |  |  |
| Liver transaminases | (To screen for, detect, evaluate and monitor acute and chronic liver inflammation, liver infection, liver disease and/or damage)  Risk factor that influence hemorrhagic, thromboembolic events | (Palareti, Leali et al. 1996, Holbrook, Schulman et al. 2012, Kim, Ozonoff et al. 2015) |
| Creatine kinase | (In particular CK-MB and CK-BB- Sign of myocardial or neurological lesion)  Risk factor that influence hemorrhagic, thromboembolic events | (Beyth, Quinn et al. 1998, Holbrook, Schulman et al. 2012) |
| Gamma-glutamyltransferase | (To evaluate for a possible liver disease or bile duct disease, sometimes to screen for or monitor alcohol abuse)  Risk factor that influence hemorrhagic, thromboembolic events | (Beyth, Quinn et al. 1998) |
| Alkaline phosphatase | (To screen for or monitor treatment for a liver disorder)  Risk factor that influence hemorrhagic, thromboembolic events | (Gage, Yan et al. 2006, Kim, Ozonoff et al. 2015) |
| Complete Blood count (CBC) | (The blood count can reveal a large number of pathologies: anemia, coagulation problem, viral infections or consumption of platelets)  Identify an hemorrhagic, thromboembolic event and risk factors | (Schulman, Kearon et al. 2005, Hirsh, Guyatt et al. 2008, Ruiz-Gimenez, Suarez et al. 2008, Konstantinides, Torbicki et al. 2014, Kearon, Ageno et al. 2016) |
| Hemostasis assessment | Identify an hemorrhagic, thromboembolic event and risk factors | (Schulman, Kearon et al. 2005, Konstantinides, Torbicki et al. 2014, Kearon, Ageno et al. 2016) |
| Other measured biological values available |  |  |
| Albumine | (To screen for and help diagnose a liver disorder or kidney disease)  Risk factor that influence hemorrhagic, thromboembolic events | (Hirsh, Guyatt et al. 2008, Efird, Mishkin et al. 2014) |
| C-reactive protein | (To identify the presence of inflammation)  Risk factor that influence hemorrhagic, thromboembolic events | (Hirsh, Guyatt et al. 2008, Lee, Park et al. 2015) |
| Myoglobin | (To determine various cardiovascular disorders)  Risk factor that influence hemorrhagic, thromboembolic events | (Gage, Yan et al. 2006, Hirsh, Guyatt et al. 2008) |
| Troponin | (May be a sign of myocardial infarction, pulmonary embolism or myocarditis)  Risk factor that influence hemorrhagic, thromboembolic events | (Gage, Yan et al. 2006, Ruiz-Gimenez, Suarez et al. 2008) |
| Creatinine and Creatinine Clearance | (To help diagnose kidney disease)  Risk factor that influence hemorrhagic, thromboembolic events | (Ruiz-Gimenez, Suarez et al. 2008, Donze, Rodondi et al. 2012) |
| Bilirubin | (To screen for or monitor liver disorders or hemolytic anemia)  Risk factor that influence hemorrhagic, thromboembolic events | (Ruiz-Gimenez, Suarez et al. 2008, Zhu, He et al. 2015) |
| Glycated hemoglobin | (To monitor a person's diabetes)  Risk factor that influence hemorrhagic, thromboembolic events | (Shireman, Mahnken et al. 2006, Lip, Frison et al. 2011) |
| Tumor markers avalaible | Risk factor that influence hemorrhagic, thromboembolic events | (Kuijer, Hutten et al. 1999, White, Beyth et al. 1999, Gage, Yan et al. 2006) |
| Prescription/medication | Identify an hemorrhagic, thromboembolic event (markers), risk factors, triggers and causal factors | (Holbrook, Schulman et al. 2012) |

**Bibliography related to the justification of extracted items**

(AHRQ), A. f. H. R. a. Q. (2017 ). " Patient Safety Indicators technical specification updates – version 7.0 (ICD 10)." from <http://www.qualityindicators.ahrq.gov/Modules/PSI_TechSpec_ICD10_v70.aspx>

Beyth, R. J., L. M. Quinn and C. S. Landefeld (1998). "Prospective evaluation of an index for predicting the risk of major bleeding in outpatients treated with warfarin." Am J Med **105**(2): 91-99.

Donze, J., N. Rodondi, G. Waeber, P. Monney, J. Cornuz and D. Aujesky (2012). "Scores to predict major bleeding risk during oral anticoagulation therapy: a prospective validation study." Am J Med **125**(11): 1095-1102.

Efird, L. M., D. S. Mishkin, D. R. Berlowitz, A. S. Ash, E. M. Hylek, A. Ozonoff, J. I. Reisman, S. Zhao, G. K. Jasuja and A. J. Rose (2014). "Stratifying the risks of oral anticoagulation in patients with liver disease." Circ Cardiovasc Qual Outcomes **7**(3): 461-467.

Gage, B. F., Y. Yan, P. E. Milligan, A. D. Waterman, R. Culverhouse, M. W. Rich and M. J. Radford (2006). "Clinical classification schemes for predicting hemorrhage: results from the National Registry of Atrial Fibrillation (NRAF)." Am Heart J **151**(3): 713-719.

Hirsh, J., G. Guyatt, G. W. Albers, R. Harrington and H. J. Schunemann (2008). "Antithrombotic and thrombolytic therapy: American College of Chest Physicians Evidence-Based Clinical Practice Guidelines (8th Edition)." Chest **133**(6 Suppl): 110S-112S.

Holbrook, A., S. Schulman, D. M. Witt, P. O. Vandvik, J. Fish, M. J. Kovacs, P. J. Svensson, D. L. Veenstra, M. Crowther and G. H. Guyatt (2012). "Evidence-based management of anticoagulant therapy: Antithrombotic Therapy and Prevention of Thrombosis, 9th ed: American College of Chest Physicians Evidence-Based Clinical Practice Guidelines." Chest **141**(2 Suppl): e152S-e184S.

Kearon, C., W. Ageno, S. C. Cannegieter, B. Cosmi, G. J. Geersing, P. A. Kyrle, A. Subcommittees on Control of, Predictive and D. Diagnostic Variables in Thrombotic (2016). "Categorization of patients as having provoked or unprovoked venous thromboembolism: guidance from the SSC of ISTH." J Thromb Haemost **14**(7): 1480-1483.

Kim, E. J., A. Ozonoff, E. M. Hylek, D. R. Berlowitz, A. S. Ash, D. R. Miller, S. Zhao, J. I. Reisman, G. K. Jasuja and A. J. Rose (2015). "Predicting outcomes among patients with atrial fibrillation and heart failure receiving anticoagulation with warfarin." Thromb Haemost **114**(1): 70-77.

Konstantinides, S. V., A. Torbicki, G. Agnelli, N. Danchin, D. Fitzmaurice, N. Galie, J. S. Gibbs, M. V. Huisman, M. Humbert, N. Kucher, I. Lang, M. Lankeit, J. Lekakis, C. Maack, E. Mayer, N. Meneveau, A. Perrier, P. Pruszczyk, L. H. Rasmussen, T. H. Schindler, P. Svitil, A. Vonk Noordegraaf, J. L. Zamorano, M. Zompatori, D. Task Force for the and C. Management of Acute Pulmonary Embolism of the European Society of (2014). "2014 ESC guidelines on the diagnosis and management of acute pulmonary embolism." Eur Heart J **35**(43): 3033-3069, 3069a-3069k.

Kuijer, P. M., B. A. Hutten, M. H. Prins and H. R. Buller (1999). "Prediction of the risk of bleeding during anticoagulant treatment for venous thromboembolism." Arch Intern Med **159**(5): 457-460.

Le Pogam, M. A., C. Quantin, O. Reich, P. Tuppin, A. Fagot-Campagna, F. Paccaud, I. Peytremann-Bridevaux and B. Burnand (2017). "Geriatric Patient Safety Indicators Based on Linked Administrative Health Data to Assess Anticoagulant-Related Thromboembolic and Hemorrhagic Adverse Events in Older Inpatients: A Study Proposal." JMIR Res Protoc **6**(5): e82.

Lee, H. H., J. M. Park, S. W. Lee, S. H. Kang, C. H. Lim, Y. K. Cho, B. I. Lee, I. S. Lee, S. W. Kim and M. G. Choi (2015). "C-reactive protein as a prognostic indicator for rebleeding in patients with nonvariceal upper gastrointestinal bleeding." Dig Liver Dis **47**(5): 378-383.

LI, I. (2013). Risk Adjustment for Measuring Health Care Outcomes. Chicago.

Lip, G. Y., L. Frison, J. L. Halperin and D. A. Lane (2011). "Comparative validation of a novel risk score for predicting bleeding risk in anticoagulated patients with atrial fibrillation: the HAS-BLED (Hypertension, Abnormal Renal/Liver Function, Stroke, Bleeding History or Predisposition, Labile INR, Elderly, Drugs/Alcohol Concomitantly) score." J Am Coll Cardiol **57**(2): 173-180.

Palareti, G., N. Leali, S. Coccheri, M. Poggi, C. Manotti, A. D'Angelo, V. Pengo, N. Erba, M. Moia, N. Ciavarella, G. Devoto, M. Berrettini and S. Musolesi (1996). "Bleeding complications of oral anticoagulant treatment: an inception-cohort, prospective collaborative study (ISCOAT). Italian Study on Complications of Oral Anticoagulant Therapy." Lancet **348**(9025): 423-428.

Ruiz-Gimenez, N., C. Suarez, R. Gonzalez, J. A. Nieto, J. A. Todoli, A. L. Samperiz, M. Monreal and R. Investigators (2008). "Predictive variables for major bleeding events in patients presenting with documented acute venous thromboembolism. Findings from the RIETE Registry." Thromb Haemost **100**(1): 26-31.

Schulman, S., C. Kearon, S. Subcommittee on Control of Anticoagulation of the, T. Standardization Committee of the International Society on and Haemostasis (2005). "Definition of major bleeding in clinical investigations of antihemostatic medicinal products in non-surgical patients." J Thromb Haemost **3**(4): 692-694.

Shireman, T. I., J. D. Mahnken, P. A. Howard, T. F. Kresowik, Q. Hou and E. F. Ellerbeck (2006). "Development of a contemporary bleeding risk model for elderly warfarin recipients." Chest **130**(5): 1390-1396.

White, R. H., R. J. Beyth, H. Zhou and P. S. Romano (1999). "Major bleeding after hospitalization for deep-venous thrombosis." Am J Med **107**(5): 414-424.

Zhu, W., W. He, L. Guo, X. Wang and K. Hong (2015). "The HAS-BLED Score for Predicting Major Bleeding Risk in Anticoagulated Patients With Atrial Fibrillation: A Systematic Review and Meta-analysis." Clin Cardiol **38**(9): 555-561.

Table S5: ATC Codes of drugs concerned by the study

| Class | ATC | Antithrombotic |
| --- | --- | --- |
| Vitamin K antagonists | B01AA04 | Phenprocoumon |
|  | B01AA07 | Acenocoumarol |
| Heparins | B01AB01 | Heparin |
|  | B01AB02 | Antithrombin III |
|  | B01AB04 | Dalteparin |
|  | B01AB05 | Enoxaparin |
|  | B01AB06 | Nadroparin |
|  | B01AB09 | Danaparoid |
| [Platelet aggregation inhibitors](https://www.whocc.no/atc_ddd_index/?code=B01AC) | B01AC04 | [Clopidogrel](https://www.whocc.no/atc_ddd_index/?code=B01AC04&showdescription=yes) |
|  | B01AC06 | [Acetylsalicylic acid](https://www.whocc.no/atc_ddd_index/?code=B01AC06&showdescription=yes) |
|  | B01AC09 | [Epoprostenol](https://www.whocc.no/atc_ddd_index/?code=B01AC09&showdescription=yes) |
|  | B01AC11 | [Iloprost](https://www.whocc.no/atc_ddd_index/?code=B01AC11&showdescription=yes) |
|  | B01AC13 | [Abciximab](https://www.whocc.no/atc_ddd_index/?code=B01AC13&showdescription=yes) |
|  | B01AC16 | [Eptifibatide](https://www.whocc.no/atc_ddd_index/?code=B01AC16&showdescription=yes) |
|  | B01AC17 | [Tirofiban](https://www.whocc.no/atc_ddd_index/?code=B01AC17&showdescription=yes) |
|  | B01AC21 | [Treprostinil](https://www.whocc.no/atc_ddd_index/?code=B01AC21&showdescription=yes) |
|  | B01AC22 | [Prasugrel](https://www.whocc.no/atc_ddd_index/?code=B01AC22&showdescription=yes) |
|  | B01AC24 | [Ticagrelor](https://www.whocc.no/atc_ddd_index/?code=B01AC24&showdescription=yes) |
|  | B01AC25 | [Cangrelor](https://www.whocc.no/atc_ddd_index/?code=B01AC25&showdescription=yes) |
|  | B01AC27 | [Selexipag](https://www.whocc.no/atc_ddd_index/?code=B01AC27&showdescription=yes) |
| Direct thrombin inhibitors | B01AE03 | [Argatroban](https://www.whocc.no/atc_ddd_index/?code=B01AE03&showdescription=yes) |
|  | B01AE06 | Bivalirudin |
|  | B01AE07 | [Dabigatran etexilate](https://www.whocc.no/atc_ddd_index/?code=B01AE07&showdescription=yes) |
| [Direct factor Xa inhibitors](https://www.whocc.no/atc_ddd_index/?code=B01AF) | B01AF01 | [Rivaroxaban](https://www.whocc.no/atc_ddd_index/?code=B01AF01&showdescription=yes) |
|  | B01AF02 | [Apixaban](https://www.whocc.no/atc_ddd_index/?code=B01AF02&showdescription=yes) |
|  | B01AF03 | [Edoxaban](https://www.whocc.no/atc_ddd_index/?code=B01AF03&showdescription=yes) |
| Other antithrombotic agents | B01AX05 | [Fondaparinux](https://www.whocc.no/atc_ddd_index/?code=B01AX05&showdescription=yes) |
