# Supplementary material for: Automatic Detection of Adverse Drug Events in Geriatric Care: Study Proposal
Source: JMIR Res Protoc. 2022 Nov 15;11(11):e40456. doi: 10.2196/40456 (PMC9709671; doi:10.2196/40456)
Supplement: Multimedia Appendix 2 [file resprot_v11i11e40456_app2.pdf]

Review: 1

Application data

Applicant(s)
Csajka, Chantal
Lang, Pierre-Olivier / Lovis, Christian / Le Pogam, Marie Annick / Beeler, Patrick

Automated detection of adverse drug events from older inpatients' electronic medical records using structured data mining and natural language processing
NRP 74: Full Proposal
Detailed evaluation

Scientific quality and originality

The project is underpinned by a very strong clinical rationale explained in a very accurate background section. The scope of the project is to identify predictors of adverse drug events associated to the use of anti-thrombotic therapies and strenghten detection tools by adding text mining to the usual modelling techniques, based on retrospective hospital data of relevant subjects. The scope and research questions addressed by the project are relevant.

The Delphi method adopted for the selection of relevant characteristics is appropriate and scientifically sound.

However, the computational algorithms proposed for the structured and unstructured data mining are absolutely unclear. In particular, the referee could not assess whether the predictive models would be based on regression modelling, or any other approach.

The sample size calculation method does not seem sufficiently clear, stable and objective. The final estimates are based on comparisons extracted from a table that presents different values, based on the arguable target of confidence intervals with a 20% width. Firstly, such confidence intervals seem too wide to guarantee the required stability required to validate the text based algorithm as opposed to the structured format. Secondly, and most importantly, the method takes into account only one aspect of reliability, i.e. sensitivity, while more complete assessment might be performed using approaches eg likelihood ratio or ROC curve, which take simultaneously into both sensitivity and specificity. Finally, traditional techniques embedding the calculation of adequate power would lead to more objective selection of minimal sample size.

Use of de-identified data may not necessarily imply no risks for privacy and data protection, given the level of detail required by the collection of individual characteristics that would allow re-identification. The challenges involved with these problems are not sufficiently addressed in the proposal. The ethical committee should consider whether patient consent would be required for the conduction of this study.

Inter- and transdisciplinarity

The project team involves experts with background in pharmacology, clinical research and medical informatics, which seems quite appropriate for the scope. However, given the methodological challenges

involved in this project, it would have been really beneficial to have a co-applicant with strong statistical background who does not show up in the list. The lack of specialistic expertise in this field is reflected by the limitations highlighted in the methodology of this proposal.

Application and implementation

The implementation plan is adequate as it involves aspects of relevant interest for patient safety eg indicators, guidelines and open source assessment forms.However, the central element involving the analysis of medical texts, and how this would be transferred to the coalface in health services, is not sufficiently explained.

Personnel and infrastructure

The personnel and infrastructure is adequate for the scope of the project.

Feasibility within the set timeframe and budget

The project seems feasible within the set timeframe and budget of the proposal.

Comment

The project aims to respond to a very relevant clinical question regarding the safety of antithrombotic drugs, proposing a targeted solution that can improve the detection tools available for adverse drug events. The proposed method of using retrospective analysis of structured as well as unstructured data can deliver a novel mixed approach that would maximise the information available. The design is supported by a Delphi method that is appropriate to identify the key elements for the proposed study. These are clear strengths that are worth to be outlined.

However, major weaknesses affect the overall score:
Computational algorithms proposed for the structured and unstructured data mining are absolutely unclear.
Sample size calculation methods do not seem sufficiently clear, stable and objective. Other methods based on summary elements eg likelihood ratios, ROC curve and power calculations would have been preferable.
Ethical challenges in the management of individual level data should have been better explained
How the analysis of medical text would be transferred in health services is not explained.

The absence of a co-applicant with strong biostatistical background is a noticeable pitfall in the composition of the project team, which might have negatively influenced the accuracy of the methodological section.
