# Supplementary material for: Automatic Detection of Adverse Drug Events in Geriatric Care: Study Proposal
Source: JMIR Res Protoc. 2022 Nov 15;11(11):e40456. doi: 10.2196/40456 (PMC9709671; doi:10.2196/40456)
Supplement: Multimedia Appendix 3 [file resprot_v11i11e40456_app3.pdf]

FNSNF
FONDS NATIONAL SUISSE
DE LA RECHERCHE SCIENTIFIQUE

www.snf.ch
Wildhainweg 3, case postale 8232, CH-3001 Berne

Division Programmes
Programmes nationaux de recherche (PNR)
Tél. +41 31 308 22 22
E-mail nfp@snf.ch

Professeur Chantal Csajka
Division de Pharmacologie clinique
Département de Médecine
Université de Lausanne
Bât. Hospitalier principal, 18.218
Avenue du Bugnon 17
CH-1011 Lausanne

Berne, le 8 décembre 2016

Requête nº 407440_167381 / 1 : décision

Madame,

Nous avons le plaisir de vous informer que le Conseil de la recherche vous alloue un subside de CHF 602'560.00 pour le projet de recherche « Automated detection of adverse drug events from older inpatients' electronic medical records using structured data mining and natural language processing ». Vous trouverez des informations complémentaires sur l'évaluation et le classement relatif de votre requête sur *my*SNF. La répartition et les conditions de l'octroi ci-jointes font partie intégrante de la décision.

Veuillez observer les dispositions du *Règlement des subsides* et du *Règlement d'exécution général relatif au règlement des subsides*. Ces documents sont à votre disposition sur le site web du FNS (cf. « Documents juridiques » ci-après). Vous trouvez également des informations détaillées sur le suivi des subsides dans les *Directives pour la gestion des projets de recherche* : www.fns.ch > le FNS > Encouragement > Documents & téléchargements > Bases juridiques. Si votre requête a été déposée en commun avec d'autres personnes, nous vous prions d'observer l'obligation d'informer les autres requérant-e-s comme mentionné aux articles 12 et 32 du *Règlement des subsides*.

Nous vous prions de remplir et nous remettre par voie électronique le formulaire en ligne "Demande de déblocage du subside" (www.mysnf.ch).

En vous souhaitant plein succès dans la réalisation de votre projet, nous vous prions d'agréer, Madame, nos salutations distinguées.

Kathrin Peter

Répartition du subside par rubrique

	Total	1ère tranche	2ème tranche	3ème tranche	4ème tranche
**Projet**					
Appareils	0	0	0	0	0
Frais de recherche	6'000	1'000	1'000	2'000	2'000
Salaires	513'201	171'843	156'854	142'517	41'987
Charges sociales	83'359	27'095	25'091	23'899	7'274
**Total**	602'560	199'938	182'945	168'416	51'261

Début: 1er janvier 2017 Durée: 48 mois

Conditions financières

Si le Conseil de la recherche devait avoir réduit votre subside de recherche, vous en trouverez les détails budgétaires dans mySNF. Veuillez s.v.p. tenir compte du nouveau « Règlement des subsides » ainsi que du « Règlement d'exécution général relatif au règlement des subsides », en particulier en ce qui concerne les salaires et les conditions concernant les doctorants et l'achat de matériel de base.

Remarque:
Si des plans visant à élaborer des produits propres ou visant à créer des liens avec des firmes de production ayant un intérêt commercial devaient exister, le groupe de recherche les mettra à jour et les rendra transparents.

Subside égalité
Les jeunes chercheuses (post)doctorantes, ainsi que les chercheuses dans les hautes écoles spécialisées ne visant pas le doctorat, qui collaborent à un taux d'occupation d'au moins 60% dans des projets financés par le FNS ont droit à un subside égalité dans le cadre de mesures visant l'encouragement de carrières. Les collaboratrices doivent être employées par une institution suisse. Les ayants-droit reçoivent au maximum CHF 1'000.- par année pour de telles mesures (c'est-à-dire par tranche de 12 mois de la durée acceptée, les tranches inférieures n'étant pas imputables). Sont considérées comme mesures de développement de la carrière, le mentorat, le coaching, les cours et ateliers d'encouragement de la carrière, les rencontres et manifestations visant à tisser un réseau, etc. Le subside égalité n'est pas destiné à payer les frais de garde des enfants.

Si les frais du subside égalité ne peuvent pas être couverts par les ressources disponibles du projet, le FNS peut déclencher un paiement de compensation sur la base d'une remarque insérée dans le cadre du rapport financier final muni des pièces justificatives correspondantes.

Informations et conditions générales

Le subside est divisé en tranches annuelles. Nous ne pouvons pas complètement exclure que les montants de celles-ci doivent être revus à la baisse si des réductions du budget de la Confédération venaient affecter les ressources du FNS. Cette réserve ne vaut pas pour le montant de la première tranche qui est assuré.

Cet octroi sera pris en compte dans le calcul du montant global de l'overhead qui sera versé à votre institution pour la couverture de frais indirects de recherche.

La demande de déblocage du subside du FNS doit être soumise en ligne via *my*SNF et remplir les conditions suivantes:

- Veuillez soumettre dans la rubrique « lay summary » un résumé des recherches prévues (cf. chiffre 8.3 du *Règlement d'exécution général* en vigueur dès le 1.1.2016).
- Pour les projets de recherche nécessitant des autorisations ou des annonces, les copies de ces documents doivent être mises à disposition.

Le FNS demande expressément aux bénéficiaires des subsides de faire figurer une version complète de leurs articles scientifiques (peer reviewed) sur le serveur de leur haute école, (cf. chiffre 11.11, *Règlement d'exécution général* ainsi que www.fns.ch -> Point recherche> Dossier > Open Access).

Autre-s bénéficiaire-s participant au projet:

- Dr. Patrick Beeler, University Hospital Zurich Centre on Aging and Mobility University of Zurich, 8037 Zürich
- Prof. Christian Lovis, Service d'Informatique médicale Hôpitaux Universitaires de Genève, 1211 Genève 9
- Dr. Marie Annick Le Pogam, Unité de Prévention Communautaire IUMSP Université de Lausanne et CHUV, 1010 Lausanne
-

Accès aux expertises externes dans *my*SNF

Le Fonds national suisse permet aux requérant-e-s d'accéder au texte intégral des expertises externes prises en compte dans l'évaluation de leur requête, à l'exception des passages qui pourraient divulguer l'identité de l'expert-e; ceux-ci sont rendus anonymes dans les expertises.

Dès le 10 décembre 2016, vous pourrez accéder aux expertises relatives à votre requête depuis *my*SNF, sous « Documents > Expertises anonymisées ».

Les organes d'évaluation du FNS s'efforcent de procéder à une appréciation globale équilibrée pour chaque requête. Les expertises externes y jouent un rôle important. L'expert-e en question n'examine en règle générale qu'une seule requête. Les organes d'évaluation du FNS doivent en revanche considérer la qualité de toutes les requêtes du semestre. Par ailleurs, les expertises sont fréquemment formulées de manière positive; parfois, elles contiennent des commentaires critiques isolés qui ne sont pas ou peu pertinents pour le travail des organes d'évaluation. Les expertises externes ne reflètent donc pas nécessairement la décision des organes d'évaluation du FNS.

Le FNS met à votre disposition les expertises à titre d'information; il n'attend pas de réponse de votre part.

Documents juridiques
(www.fns.ch >Encouragement > Documents & téléchargements > Bases juridiques)

- Règlement des subsides
- Règlement d'exécution général relatif au règlement des subsides

Indication des voies de droit
Conformément à l'article 13 de la loi du 14 décembre 2012 sur l'encouragement de la recherche et de l'innovation (RS 420.1), la présente décision peut faire l'objet d'un recours, dans un délai de 30 jours après sa notification, auprès du Tribunal administratif fédéral, case postale, 9023 St-Gall.

Le mémoire de recours indique les conclusions, motifs et moyens de preuve et porte la signature du ou de la recourant-e ou de son mandataire.

La décision attaquée et les pièces invoquées comme moyens de preuve, lorsqu'elles se trouvent en la possession du ou de la recourant-e, doivent être annexées à l'envoi.
